# Towards further understanding the applications of endophytes: enriched source of bioactive compounds and bio factories for nanoparticles

**DOI:** 10.3389/fpls.2023.1193573

**Published:** 2023-07-10

**Authors:** Nisha Choudhary, Naveen Dhingra, Amel Gacem, Virendra Kumar Yadav, Rakesh Kumar Verma, Mahima Choudhary, Uma Bhardwaj, Rajendra Singh Chundawat, Mohammed S. Alqahtani, Rajarshi Kumar Gaur, Lienda Bashier Eltayeb, Waleed Al Abdulmonem, Byong-Hun Jeon

**Affiliations:** ^1^ Dept of Biosciences, School of Liberal Arts and Sciences, Mody University of Science and Technology, Lakshmangarh, Sikar, Rajasthan, India; ^2^ Department of Agriculture, Medi-Caps University, Pigdamber Road, Rau, Indore, Madhya Pradesh, India; ^3^ Department of Physics, Faculty of Sciences, University 20 Août 1955, Skikda, Algeria; ^4^ Department of Life Sciences, Hemchandracharya North Gujarat University, Patan, Gujarat, India; ^5^ Department of Biotechnology, Noida International University, Noida, U.P., India; ^6^ Radiological Sciences Department, College of Applied Medical Sciences, King Khalid University, Abha, Saudi Arabia; ^7^ BioImaging Unit, Space Research Centre, University of Leicester, Leicester, United Kingdom; ^8^ Department of Biotechnology, Deen Dayal Upadhyaya (D.D.U.) Gorakhpur University, Gorakhpur, Uttar Pradesh, India; ^9^ Department of Medical Laboratory Sciences, College of Applied Medical Sciences, Prince Sattam Bin AbdulAziz University- Al-Kharj, Riyadh, Saudi Arabia; ^10^ Department of Pathology, College of Medicine, Qassim University, Buraidah, Saudi Arabia; ^11^ Department of Earth Resources and Environmental Engineering, Hanyang University, Seoul, Republic of Korea

**Keywords:** nano particle, plant growth promotion, larvicidal, bioactive component, endophytes

## Abstract

The most significant issues that humans face today include a growing population, an altering climate, an growing reliance on pesticides, the appearance of novel infectious agents, and an accumulation of industrial waste. The production of agricultural goods has also been subject to a great number of significant shifts, often known as agricultural revolutions, which have been influenced by the progression of civilization, technology, and general human advancement. Sustainable measures that can be applied in agriculture, the environment, medicine, and industry are needed to lessen the harmful effects of the aforementioned problems. Endophytes, which might be bacterial or fungal, could be a successful solution. They protect plants and promote growth by producing phytohormones and by providing biotic and abiotic stress tolerance. Endophytes produce the diverse type of bioactive compounds such as alkaloids, saponins, flavonoids, tannins, terpenoids, quinones, chinones, phenolic acids etc. and are known for various therapeutic advantages such as anticancer, antitumor, antidiabetic, antifungal, antiviral, antimicrobial, antimalarial, antioxidant activity. Proteases, pectinases, amylases, cellulases, xylanases, laccases, lipases, and other types of enzymes that are vital for many different industries can also be produced by endophytes. Due to the presence of all these bioactive compounds in endophytes, they have preferred sources for the green synthesis of nanoparticles. This review aims to comprehend the contributions and uses of endophytes in agriculture, medicinal, industrial sectors and bio-nanotechnology with their mechanism of action.

## 1 Introduction

**Figure 1:**
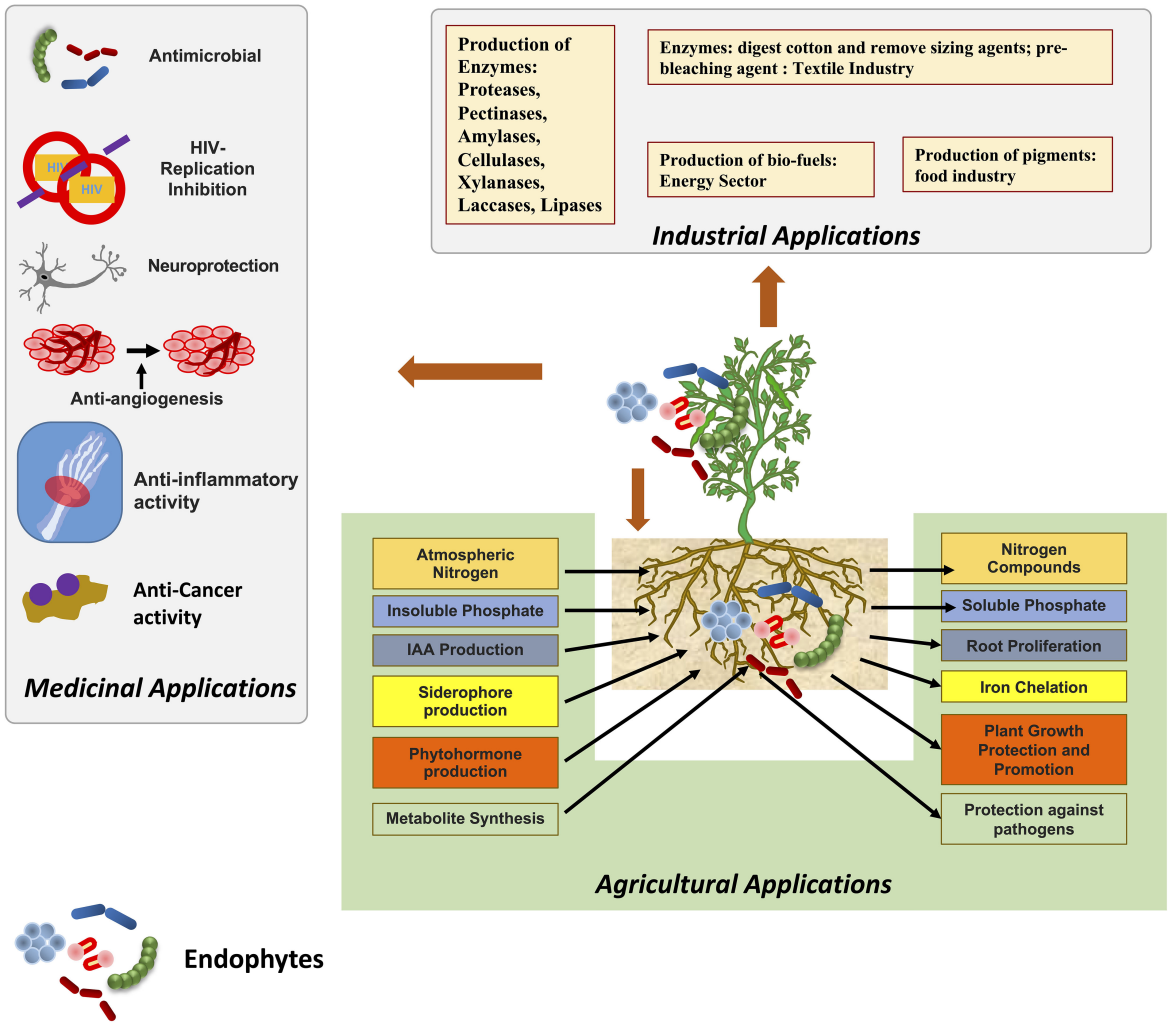
Endophytes and their applications in agriculture, industrial and medical fields.

According to the report published by the IPCC (2018), the probability of limiting the effects of global warming to 1.5°C is majorly determined by the cumulative emission of carbon dioxide (CO_2_) and future non-CO_2_ radiative forcing. The devastating impact of climate change can be observed in sustainable agriculture systems and overall agriculture productivity (Verma et al., 2022). Agricultural activities in the 21^st^ century are largely depending on the extensive use of fertilizers, pesticides, fungicides etc. and also sometimes involve the over-irrigation and use of high-yielding crop varieties (Hegazy et al., 2017). Such practices have a negative impact on the environment and lead to low fertility of the soil by decreasing the symbiotic association of fungal and bacterial communities in the soil. Such practices also lead to groundwater pollution due to the leaching out of nitrogen and phosphorus from the soil into the groundwater (Rafi et al., 2019). In a similar vein, many biotic factors, such as bacteria, fungi, viruses, weeds, insects, and nematodes, are major constraints of stress that tend to increase the reactive oxygen species that affect the physiological and molecular functioning of plants and also lead to the decrease in crop productivity (Chaudhary et al., 2022). In addition, the increased temperatures, atmospheric CO_2_ levels, and precipitation patterns also affect agricultural production and insect infestations (Skendžić et al., 2021). In order to reduce the negative impact of such practices and to maintain the fertility of the soil, various eco-friendly farming techniques are employed such as the inculcation of microorganisms as fertilizers encompassing nutrient mobilizing capacity (Gamez et al., 2019). The symbiotic relationship between plants and microorganisms exerts various benefits on plants such as an increase in height and weight, the high nutritional value of plants etc. It results in increasing crop yield, nutrient cycling and fertility of the soil (Card et al., 2021). A single plant is colonised by a large number of microbes, these microbes can be termed epiphytes and endophytes. Endophytes, symbiotic bacterial and fungal communities, are present in intercellular and intracellular spaces of plant parts, such as stems, roots, leaves, etc. (Oukala et al., 2021). Endophytes are also accommodated by weeds inflorescences, petioles, buds, and dead and hollow hyaline cells of plants, fruits and seeds (Lonkar and Bodade, 2021). Either the full or some part of the life cycle of endophyte microbes occur inside the host plant, without causing any negative impact on the plant (Joo et al., 2021). Endophytic associations are reported in various types of plants such as soybeans, chickpeas, cowpeas, sunflower, pearl millet, rice, maize, mustard, sugarcane, cotton, tomatoes, etc (Fadiji and Babalola, 2020). Endophytes are classified on the basis of their biological nature, mode of transmission, and diversity into two classes: transient endophytes and true endophytes (Sharma et al., 2021). In addition, on the basis of their association with host plants, endophytes are classified as obligate and facultative endophytes. Endophytes that spread among plants vertically and completely rely on plant metabolism for survival are referred to as “obligate endophytes,” whereas endophytes that enter plants from neighbouring soil or environment and only partially rely on the host plant, completing only some part of the host plant’s lifecycle, are referred as “facultative endophytes” (Khare et al., 2018). The endophytic world has gained popularity among researchers due to its significant contributions as it produces various types of bioactive compounds that play important roles in various industries such as agricultural, pharmaceutical, medical, and biotechnological industries (**Figure 1**) (Latz et al., 2018; Tiwari et al., 2023).

Endophytes have a significant impact on the health of their hosts, including their ability to absorb nutrients, produce phytohormones, and reduce the damage caused by pathogens through antibiosis, the generation of lytic enzymes, the activation of secondary metabolites, and the activation of hormones (Chaudhary et al., 2022). However, the complete elucidation of total metabolites produced by endophytes, their functions, protein-protein interactions, and the factors which influence the interaction between fungal, and bacterial endophytes with different plants is still in inferencing.

Nowadays, both fungal and bacterial endophytes are important in the industrial, pharmacological, and biotechnological sectors. This is because they produce different types of metabolites that are used as antitumor, antiviral, and antimicrobial agents, plant growth promoters, bio-control agents, stress tolerance of plants, and immunosuppressants. They also produce different types of compounds that make them good antibiotic, anti-diabetic, and antioxidant agents (Gouda et al., 2016). Several researchers have demonstrated the potential of these endophytes in the synthesis of novel nanomaterials and the role of microbial endophytes in agriculture (Yadav et al., 2020), (Dhara et al., 2023).

In the present review, the authors have emphasized the importance of both fungal and bacterial endophytes in industries, pharmaceuticals and biotechnology. This study addressed recent research on endophytes in order to address a gap in the field and give a detailed application of the metabolites or bioactive components that have been isolated from endophytes and their potential application to bio-nanotechnology. In addition, this review has emphasised on the importance of endophytes-mediated synthesis of nanoparticles as well as their applicability in a variety of sectors.

## 2 Various approaches for screening bioactive compounds in endophytic culture

**Figure 2:**
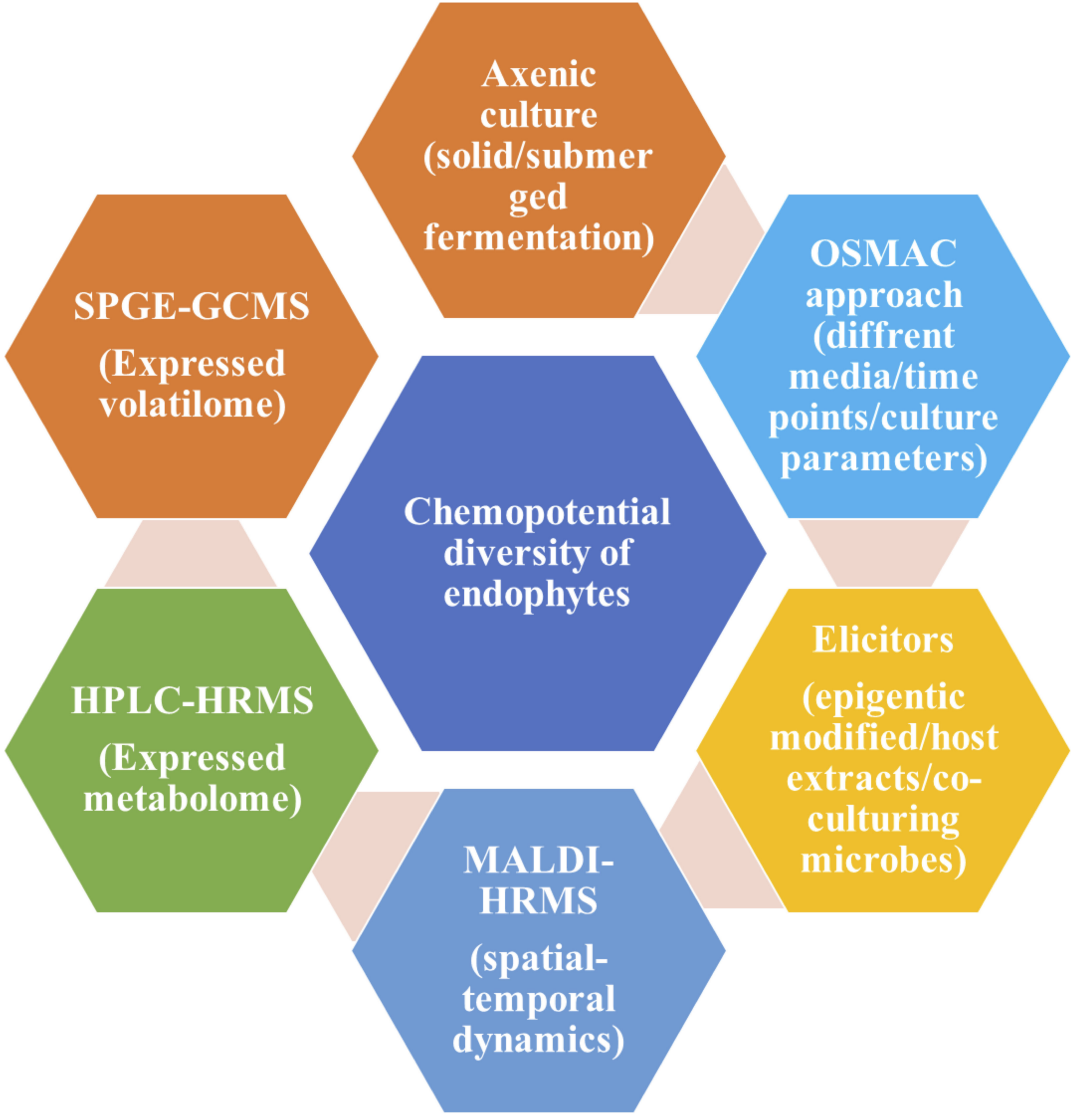
Schematic diagram for screening of various bioactive compounds from endophytic culture adopted from (Mishra et al., 2022).

From the various pieces of literature, it has been proven that there are various approaches for the screening of bioactive compounds from the culture of endophytes like axenic culture, OSMAC approach, and elicitors (Gakuubi et al., 2021). All these three approaches play an important role in the activation of cryptic gene clusters in the endophyte’s genome. In addition to this, there are several instrument-based approaches like solid-phase microextraction-gas chromatography-mass spectroscopy (SPME GCMS), high-performance liquid chromatography high-resolution mass spectroscopy (HPLC-HRMS) and matrix-associated laser desorption ionization-HRMS (MALDI-HRMS). SPGE-GC-MS helps in the screening of volatile compounds especially signalling compounds secreted by the endophytes. HPLC-HRMS helps in the screening for bioactive compounds mainly antimicrobial and anti-cancerous. MALDI-HRMS, the technique could help in the screening of the distribution and release of target compounds in host plants’ apoplast (Mishra et al., 2022). **Figure 2** is showing a schematic diagram for various approaches for screening bioactive compounds from endophytic culture.

## 3 The mechanism employed by endophytes in plant growth promotion and protection

Endophytes protect plants by employing two types of mechanisms: indirect and direct. In direct mechanism, endophytes directly promote plant cell elongation and proliferation by producing phytohormones, indole-3-acetic acid (IAA), siderophores, 1-aminocyclopropane-1-carboxylic acid, phosphate and potassium solubilization antibiosis, and by suppressing the pathogens, etc.

In addition, endophytes promote the capacity to convert atmospheric nitrogen into ammonia, which is required for the synthesis of proteins and nucleic acids and provides tolerance against salt and drought by synthesizing sugar molecules (Muthu Narayanan et al., 2022 **).** Several studies have highlighted the role of endophytic fungi in mitigating biotic and abiotic stresses, making them an essential component of climate-smart and sustainable agriculture (Verma et al., 2022), (Tyagi et al., 2022). The endophytes are known to produce various types of siderophores such as carboxylate, catecholate, phenolates, and hydroxamates. These siderophores perform a variety of functions, including the biocontrol of phytopathogens by limiting the pathogens’ ability to absorb iron, the reduction of heavy metal toxicity, and the induction of induced systemic resistance (ISR) (Chaudhary et al., 2022), (Gómez-Godínez et al., 2023). Several microbial species such as *Rhodococcus spp, Bacillus spp, Enterobacter spp*, Methylobacterium *spp, Pseudomonas fluorescens, Pseudomonas putida, Pantoea ananatis* and *Pantoea agglomerans*, etc. are positively shown to produce siderophores (Karuppiah et al., 2022; Singh et al., 2022). Endophytes emit different organic acids such as malic, gluconic, and citric acids that convert insoluble soil phosphate (apatite, fluorapatite and hydroxyapatite) into soluble orthophosphates by chelating cations attached to the phosphate (Fadiji and Babalola, 2020), (Yadav et al., 2018). Various bacterial and fungal endophytes have been reported to date for phosphate solubilisation activity, phosphate solubilisation, phytohormone production and nitrogen fixation activity (**Supplementary Table 1**). Endophytic associative nitrogen-fixing microbes are superior to rhizosphere microorganisms in terms of their ability to enable plant life to flourish in nitrogen-deficient soil and to support the overall health and growth of plants (Afzal et al., 2019). *Curvularia geniculata*, a dark septate root endophytic fungus isolated from the roots of *Parthenium hysterophorus*, is known to stimulate the growth of plants by solubilising phosphorus (P) and producing phytohormones (Mehta et al., 2019). Colonisation by the endophytic fungus *Serendipita indicia* enhances nutrient uptake and helps maintain ionic homeostasis by limiting the passage of sodium (Na^+^) and potassium (K^+^) ions in plants and enhancing gene transcription, both of which play important roles in Na+ and K^+^ homeostasis (Tyagi et al., 2022). Endophytes like *Colletotrichum, Pseudomonas, Bacillus. Herbaspirillum, Alcaligenes, Streptomyces, Piriformospora indica, Sebacina vermifera*, and *Penicillium* have gained particular interest amongst others because of the propensity to produce phytohormones such as auxins, gibberellins (GA), cytokinins and ethylene that favour improved plant development under harsh conditions (Burragoni and Jeon, 2021), (Omomowo et al., 2023). It has been found that the *pestalotiopsis microspore* produces pestalotin analogue, a metabolite with gibberellin activity that promotes faster germination (Li et al., 2018). Likewise, *Cladosporium sphaerospermum*, an endophyte of Glycine max, is responsible for the production of gibberellic acid, which is known to encourage the growth of rice and soybean plants (Omomowo et al., 2023).

The indirect mechanism adopted by endophytes promotes the plant growth by enhancing the plant defence system using various mechanisms like plant resistance induction, environmental stress tolerance, predation and hyperparasite, stimulation of secondary metabolites in plants etc (Tian et al., 2008). When a plant is under attack from a biotrophic pathogen, signalling molecules like salicylic acid (SA) and associated pathogenesis-related (PR) proteins act to induce “systemic acquired resistance” (SAR), which in turn triggers “local resistance” by producing a hypersensitivity reaction (HR) in the infected and surrounding areas of the plant (Muthu Narayanan et al., 2022). For instance, pre-treatment of *Pisum sativum* seeds with *Pseudomonas fluorescens* (OKC) and *Trichoderma asperellum* (T42) prevents powdery mildew disease by stimulating the defence response by upregulation of phytohormone, SA, and PR-1 protein (Patel and Saraf, 2017).

Induced system resistance (ISR) is the second defence mechanism plants use to fend against infections (Qin et al., 2021). By triggering the release and transport of signalling molecules like JA and associated PR proteins to the affected areas, it helps plants defend themselves against necrotrophic diseases. Neither the pathogenic virus nor its replication is directly hindered by the ISR approach. On the contrary, it reinforces the plants’ inherent physical or chemical defences (Muthu Narayanan et al., 2022). For example, the modulation of signalling pathways by JA and its product JA-isoleucine (JA-Ile) hormone is often achieved by the utilisation of abscisic acid (ABA) or ethylene (necrotrophic pathogens defender) (Muthu Narayanan et al., 2022). ISR is induced by *Bacillus subtilis* PTA-271 and *Pseudomonas fluorescens* PTACT2 to prevent canker and grey mould disease caused by *Pseudomonas syringae* Pst DC3000 and *Botrytis cinerea*, respectively, in the Arabidopsis plants. Infected plant leaves provide evidence of their antagonistic impact through an increase in JA and ABA (Nguyen and Nguyen, 2020).

In antibiosis, various secondary metabolites such as lipopeptides antibiotics, amino acid-rich peptides (neomycin), and cyclic cationic lipopeptides are produced by different endophytes that serve as biocontrol agents as they exhibit antifungal, antibacterial, and nematocidal activities against phytopathogens (Muthu Narayanan et al., 2022). For instance, to protect the leaves of the *W. somnifera* plant from infection by *A. alternata*, endophytic bacteria like *B. amyloliquefaciens* and *P. fluorescens* enhance callose deposition in guard cells (Mishra et al., 2018). Epichloe is a monophyletic genus of filamentous fungi that develop everlasting symbioses with cool-season grasses (Card et al., 2021). Epichloe fungal endophytes not only boost plant immunity against chewing insects by creating protective alkaloids, but they also promote plant immunity by increasing endogenous defence responses mediated by the jasmonic acid (JA) pathway (Card et al., 2021). The foliar endophytic fungus *Colletotrichum tropicale* isolated from *T. cocoa* protects the cocoa tree from black pod disease caused by *Phytophthora* spp. by upregulating genes related to cellulose and lignin deposition and host cell wall hardening (Sadoral and Cumagun, 2021). *Bacillus atrophaeus* and *Bacillus mojavensis*, isolated from the *Glycyrrhiza uralensis* (Licorice) plant, have antifungal activity due to the presence of various compounds such as 1,2-bezenedicarboxyl acid, methyl ester, decanodioic acid, and bis (2-ethylhexyl) ester (Mohamad et al., 2018). The antibacterial activity of Aspergillus sp., endophyte, isolated from the *Bauhinia guianensis* plant has been reported due to the presence of fumigaclavine C and pseurtotin C (Wen et al., 2022). In another study, the protein bacillomycin D was produced by *B. amyloliquefaciens*, which was shown to have antagonistic action against the fungus *F. graminearum* (Gu et al., 2017).

## 4 Endophytic bioactive components of medicinal significance

**Figure 3:**
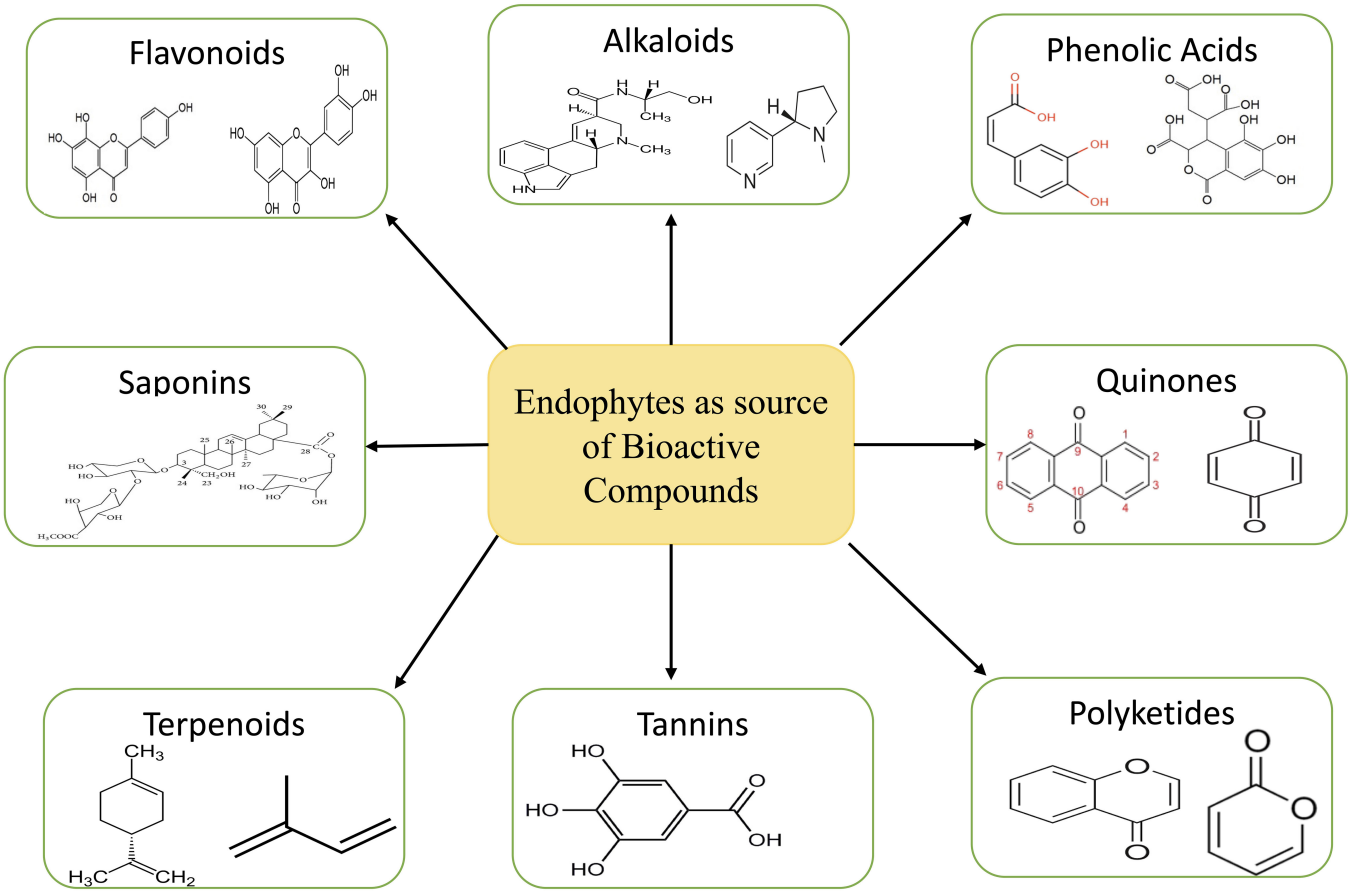
Bioactive compounds extracted from endophytes.

The ecosystem is a treasure trove of medicinal plants that contain chemicals that have the potential to serve as a substitute for medications produced synthetically (Michel et al., 2020). The major problem with collecting these compounds from medicinal plants is that there aren’t very many of them, while the demand is high. This leads to overexploitation, which in turn reduces plant population (Palanichamy et al., 2018). In order to fulfil the large-scale production of these compounds, alternative approaches like tissue culture, semi-synthesis, exploitation of endophytes for these compound syntheses, heterologous production, etc. are adopted (Sandargo et al., 2019). Endophytes present in plants are considered a treasure house of various bioactive compounds such as tannins, alkaloids, terpenoids, benzopyranones, quinones, polyketides, chinones, saponins, flavonoids, phenolic acids, steroids, xanthones etc. (**Figure 3**) (Kumari et al., 2020; Vaid et al., 2020). These bioactive compounds are known to have various uses in the medical sector.

Taxol (Paclitaxel) is a diterpene alkaloid which is produced in Taxus sp. by different endophytes such as- *Taxomyces andreanae, Fusarium solani, Metarhizium anisopliae* and many others which exhibit anticancer (antitumor) activity (**Table 1**). Taxol acts as a mitotic inhibitor and causing microtubules to break down at the time of cell division. Whereas other bioactive compounds such as- Brefeldin-A, Phomopsin-A/B/C, Cytosporone-B/C, Terpene, and cryptocandin produced in different plants *Quercus variabilis Blume, Excoecaria agallocha L., Allamanda cathartica L.* and *Tvipterigeum wilfordii Hook. f.* by different endophytes such as *Cladosporium* sp.*, Phomopsis* sp., and *Cryptosporiopsis quercina* are known to have antimicrobial, antifungal, antibacterial and antimycotic activities respectively. Similarly, Lovastatin is produced in the *Solanum xanthocarpum* plant by *Phomopsis vexans* endophyte known to have blood cholesterol-lowering properties (Parthasarathy and Sathiyabama, 2015). Lovastatin inhibits the HMG-CoA reductase enzyme (hydroxyl methylglutaryl coenzyme A reductase), which plays an important role in the regulation of the rate-limiting step of cholesterol biosynthesis as this enzyme catalyses the conversion of HMG-CoA to mevalonate, in a competitive manner (Bhargavi et al., 2018). Ligustrazine which is also known as TMP (Tetra methylpyrazine) can stimulate neuronal differentiation by controlling Topoisomerase IIβ epigenetic activity (Lin et al., 2022). It safeguards against the oxygen-glucose deprivation-induced degeneration of neurons, encourages the migration of brain progenitor/precursor cells and also inhibits H_2_O_2_-induced apoptosis in bone marrow-derived mesenchymal stem cells by controlling the PI3K/Akt and ERK1/2 signalling pathways. 3-Nitropropionic acid and tenuazonic acid exhibit strong antitubercular effects on *M. tuberculosis* H37Ra by disrupting the isocitrate lyase enzyme pathways required for the metabolism and virulence of the pathogen (Adeleke and Babalola, 2021). HupA (huperzine A) acts as a cholinesterase inhibitor (ChEI) which function to decrease the breakdown of acetylcholine and is used in dementia and Alzheimer’s treatment (Dou et al., 2018). Likewise; DPT (Deoxypodophyllotoxin) cyclolignan compound, isolated from various plants accompanied by endophytes (**Table 1**). The anti-cancer effect of DPT on colorectal cancer cells through induction of apoptosis, by destabilization of microtubules, activation of mitochondrial apoptotic pathway via regulation of B-cell lymphoma 2 (Bcl-2) family proteins, (decreasing Bcl-xL and increasing Bcl-2 associated X (BAX)) and suppression of tumorigenesis have been reported recently (Gamage et al., 2019). In addition, bilobalide, sesquiterpene tri-lactone, obtained from *Ginkgo biloba*, could be a potential therapeutic agent for brain ischemia and neurodegeneration due to its upregulation of mitochondrial DNA-encoded COX III subunit of cytochrome c oxidase and the ND1 subunit of NADH dehydrogenase genes. Both genes are involved in neuroprotection through the preservation of mitochondrial functions and hindrance in apoptosis. Nowadays the prodrug approach is often used to combat pharmacokinetic, pharmaceutical, and thermodynamic barriers that limit the inculcation of new drugs. The *influenza virus* is a deadly virus causing severe damage to human beings. Neuraminidase (NA) inhibitor drugs nowadays are used to treat influenza infections. But due to its antigenic drift and antigenic shift, the *influenza virus* is continuously evolving and may become resistant to previous drugs. Recently, cyclosporine A (CsA) and its analogues have been reported for antiviral activity against influenza A and B strains (Ma et al., 2016). Cyclosporine is a natural product and can be produced by endophytes (**Supplementary Table 1**). Likewise, *Human cytomegalovirus* (hCMV) encodes a 256 amino acid serine protease which is responsible for capsid assembly, an essential process for herpes virus production. Cytonic acids A and B, protease inhibitors, obtained from endophytic fungi *Cytonaema* sp., prevent the development of infectious herpes viruses by blocking the assembly (Tiwari et al., 2023).

**Table 1:** Plant growth promotion activities of endophytes and their host plants.

Host Plant	Endophyte	Plant Growth Promotion Activity	References
*Ephedra pachyclada*	Fifteen Fugal endophyte species	Ammonia production, phosphate solubilization (PS), and IAA production	(Khalil et al., 2021)
*Pulicaria incisa*	Fifteen bacterial endophyte species	Ammonia production, phosphate solubilization, and IAA production	(Fouda et al., 2021)
*Fagonia mollis Delile and Achillea fragrantissima*	Thirteen bacterial endophyte species	Ammonia production, PS, and indole acetic acid production	(ALKahtani et al., 2020)
*Piper nigrum*	Twelve bacterial endophyte species	PS, IAA production, siderophore production	(Jasim et al., 2013)
*Teucrium polium*	Seven bacterial and five fungal endophyte species	Ammonia production and PS	(Hassan, 2017)
*Salicornia europaea*	Thirty-two bacterial endophyte species	PS, IAA production	(Zhao et al., 2016)
*T. apollinea Moringa peregrina*	Five bacterial endophyte species	Plant growth of soybean, IAA production	(Asaf et al., 2017)
*Agave tequilana*	Eleven bacterial endophyte species	N_2_ fixation, IAA production and PS	(Martínez-Rodríguez et al., 2014)
*Tephrosia apollinea*	Thirteen bacterial endophytes	IAA production and gibberellins production	(Khan et al., 2014)
*Cucumis sativus*	*Paecilomyces formosus*	Gibberellins and IAA	(Khan et al., 2012)
*Vigna unguiculata*	*Azotobacter, Azospirillum, Rhizobium*	N_2_ fixation and phytohormone production	(Arafa and El-Batanony, 2018)
*Oryza sativa*	*Sphingopyxis granuli* and *Pseudomonas aeruginosa*	Nitrogen metabolism	(Battu et al., 2017)
*Cucumis sativus*	*Phoma glomerata, Penicillium* sp.	Gibberellins and IAA	(Waqas et al., 2012)
Glycine max	*Streptomyces* sp. NEAU-S7GS2	Biocontrol and biofertilizer	(Liu et al., 2019)
*Morus alba*	*Bacillus subtilis 7PJ-16*	Antifungal, biofertilizer and biocontrol	(Xu et al., 2019)
*Triticum aestivum L.*	*Bacillus* sp. *strain WR11*	Abiotic stress alleviation	(Chen et al., 2020)
Glycine max	*Methylobacterium, Rhizobium*	Metabolite synthesis	(Hamayun et al., 2017)
*Cajanus cajan (L.) Mill sp*	*Fusarium* sp.*, Neonectria* sp.	Cajaninstilbene acid	(Fuller et al., 2019)
*Helianthus annuus L.*	*Bacillus* sp.*, Achromobacter* sp.*, Alcaligenes* sp.	ABA, JA and phosphate solubilization	(Forchetti et al., 2007; Shahid et al., 2015)
*Phragmites karka*	*Mangrovibacter* sp. strain MP23	Uptake of nutrients, N_2_ fixation (NF) and oxidative stress	(Behera et al., 2016)
*Oryza sativa*	*Azotobacter*	Siderophore production, NF and phosphate solubilization	(Banik et al., 2016)
*Indigofera argentea*	*Enterobacter* sp. SA187	Oxidative stress and antimicrobial compounds production	(Andrés-Barrao et al., 2017)
Glycine max	*Sphingomonas* sp. LK11	IAA production, phytoremediation and PS	(Asaf et al., 2018)
*Ammodendron bifolium*	*Bacillus mojavensis*, *Bacillus* sp.	IAA production, 1-aminocyclopropane-1-carboxylic acid deaminase activity, PS and NF	(Maheshwari et al., 2020)
*Solanum lycopersicum*	*Azospirillum, Pseudomonas*	PS and NF	(Bergna et al., 2018)
*Oryza sativa L.*	*Bacillus paralicheniformis*	NF	(Annapurna et al., 2018)
*Pennisetum sinense Roxb*	*Klebsiella variicola GN02*	NF	(Lin et al., 2019)
*Pellaea calomelanos*	*Pseudarthrobacter phenanthrenivorans MHSD1*	Siderophore production and NF	(Tshishonga and Serepa-Dlamini, 2020)
*Orchid doritaenopsis*	*Mycobacterium mya-zh01*	Seed germination	(Pan et al., 2020)
*Corchorus olitorius*	*Micrococcus luteus*, *Kocuria* sp.	Siderophore production and IAA production	(Haidar et al., 2018)
*Zygophyllum simplex*	*Paenibacillus* sp. JZ16	Biotic and abiotic stress tolerance	(Eida et al., 2020)
Triticum aestivum	*Cladosporium herbarum*, *Azotobacter chroococcum and Bacillus circulans*	PS, NF and biocontrol agent	(Larran et al., 2016)
*Vicia faba L., Secale cereale L., Zea mays L., Triticum aestivum L., Equisetum arvense L. and Arctium lappa L.*	*Novosphingobium, Delftia, Achromobacter, Stenotrophomonas, Rhizobium, Brevundimonas, Variovorax, Comamonas, and Collimonas*	Siderophore production, IAA production, PS & NF	(Woźniak et al., 2019)
*Nicotiana tabacum*	*Pseudomonas* spp.	Trace metal tolerance, IAA production, NF, siderophore production, 1-aminocyclopropane-1-carboxylic acid deaminase activity and PS	(Ghanta et al., 2011)
*Tephrosia apollinea*	*Sphingomonas* sp.,	Drought tolerance	(Asaf et al., 2017; Asaf et al., 2018)
*Urochloa ramosa*	*Curtobacterium* sp.*, Microbacterium* sp.*, Methylobacterium* sp.*, Bacillus amyloliquefaciens, Actinobacteria*	PS, biocontrol and auxin production	(Asaf et al., 2017; Asaf et al., 2018; Verma and White, 2018)
*Avicennia marina*	*Micrococcus yunnanensis*	Siderophore production, IAA production and ammonia production	(Soldan et al., 2019)
*Lilium lancifolium*	*Paenibacillus polymyxa*	Siderophore production, IAA production, ammonia production, 1-aminocyclopropane-1-carboxylic acid deaminase activity, PS, NF and biocontrol	(Khan et al., 2020)
Cool-season grasses of the sub-family *Pooideae*	Epichloe fungal endophytes	Increased the plant production of SA and enhanced the expression levels of plant genes of synthesis and response to the SA hormone	(Bubica Bustos et al., 2022)
Wheat plant (*T. aestivum*)	*Alternaria, Cladosporium, Penicillium, Cryptococcus spps*	Reduction in pathogen infection	(Rojas et al., 2020)
Apple orchid (*Malus domestica*)	*Penicillium, Fusarium, Chaetomium*	As a bio-control agents	(Liu et al., 2020)
Crucifers (*Brassica oleracea, B. rapa & Raphanus sativus*)	More than 15 fungal endophytes including *Trichoderma, Fusarium* etc	Antagonist effect (AGE) against the pathogenic fungi	(Chen et al., 2016)
Tea plant (*Camellia sinensis*)	*C. gleosporioides*	AGE against the pathogenic fungi	(Rabha et al., 2014)
Tomato plant (*Solanum lycopersicum*)	Species belonging to *Alternaria, Curvularia, Fusarium, Trichoderma* and many more	Antagonistic activities against the pathogenic nematodes	(Bogner et al., 2016)
*Chrysanthemum (Dendrobium* sp.*)*	Species of *Fusarium* md *Colletotrichum*	Increased IAA production and	(Shah et al., 2019)
Maize (Zea mays)	Around 8-9 fungal endophytes including *Acremonium, Cladosporium*,	Biological control against soybean pathogens	(de Souza Leite et al., 2013)
Onion (*Allium longicuspis*)	Alternaria, Fusarium and several species of Aspergillus	Protection against the pest *Thrips tabaci*	(Muvea et al., 2014)
Periwinkle (*Catharanthus roseus*)	*Macrophomina, Fusarium, Nigropspora* and *Colletotrichum*	Production of extracellular enzymes	(Ayob and Simarani, 2016), (Ayob and Simarani, 2016)
Neem (*Azadirachta indica*)	*Xylaria, Chloridium, Fusarium, Verticillium, Colletotrichum, Trichoderma, Curvularia*	Secretion of bioactive compounds	(Chutulo and Chalannavar, 2018)
Rapeseed (*Brassica napus*)	Botrytis, Rhizoctonia, Rhizopus, *C.gloesporioides*, Aspergillus, Phoma, Alaternaria, Penicillium	AGE against the pathogenic fungi	(Zhang et al., 2014)
Orchid (*Vanda cristata*)	*Fusarium sps*	PGR activities	(Liu-Xu et al., 2022)
*Morchella tomentosa (leaves)*	*Trichoderma longibrachiatum, Syncephalastrum racemosum*	Antagonistic effect (group of fungi) and ACA	(Ibrahim et al., 2017)
*Euphorbia prostata*	*Byssochlamys spectabilis Alternaria* sp.	ABA	(Khiralla et al., 2016)
*Vernonia amygdalina leaves*	*Cladosporium cladosporioides 2*	ACA	(Khiralla et al., 2016)
*Cephalotaxus hainanensis Li (all parts)*	*Phomopsis quercella, Colletotrichum boninense, Neonectria macroconidialis, Xylaria* sp.	AMA	(Yang et al., 2015)
Bauhinia forficata	*Acremonium curvulum, Asp. ochraceus, Gibberella fujikuroi, Myrothecium verrucaria and Trichoderma piluliferum, Penicillium glabrum*	AMA	(Bezerra et al., 2015)

## 5 Endophytes of agricultural significance

**Figure 4:**
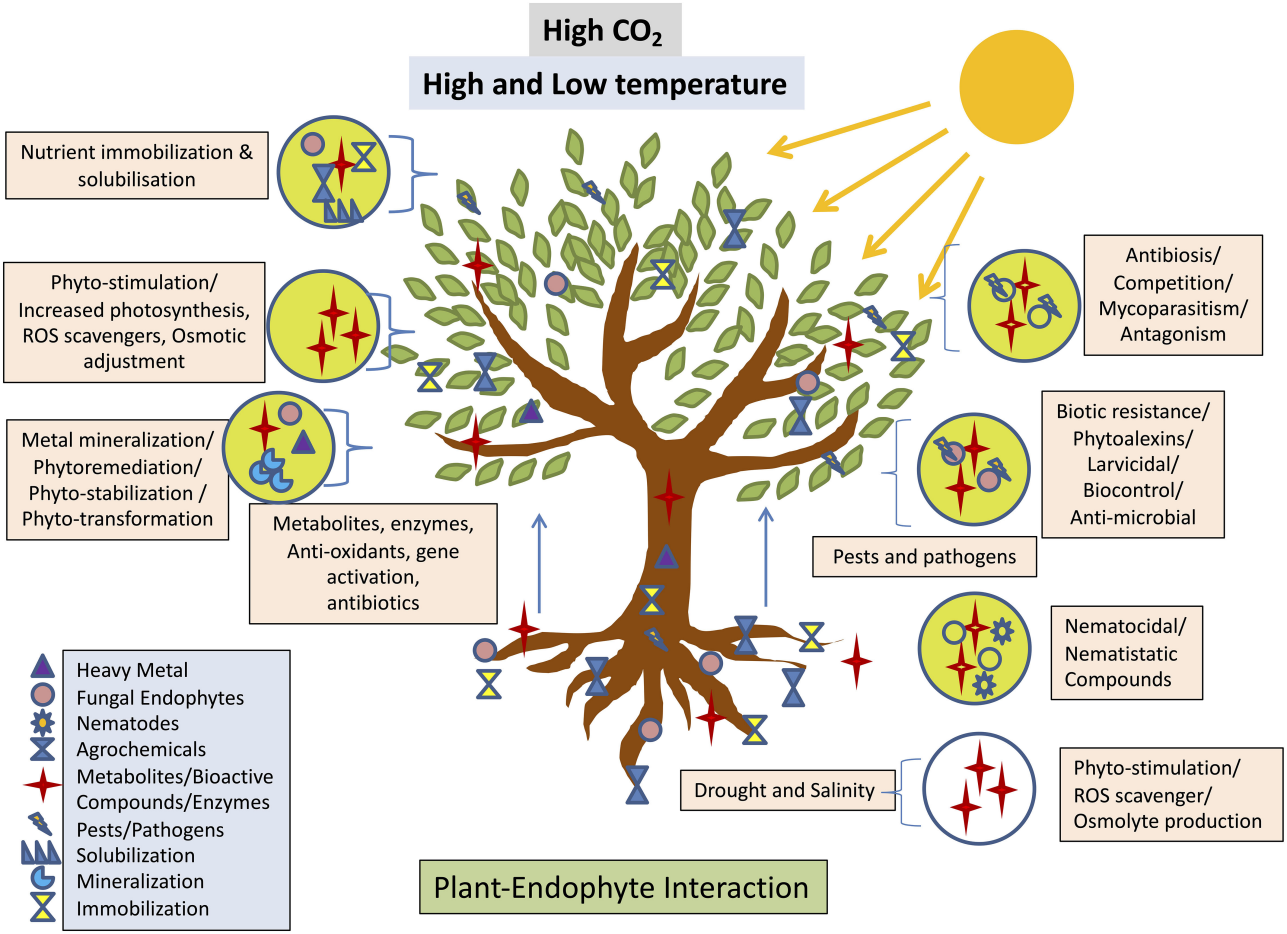
Interaction between fungal endophyte and plant expediates Phyto-stimulation conferring the stress response adopted from (Verma et al., 2022).

Endophytes exhibits numerous plant growth-promoting activities such as phosphate solubilization, siderophore production, IAA production, nitrogen fixation, ammonia production, etc. (Hashim et al., 2020; Zamin et al., 2020) *Piriformospora indica*, an endophytic basidiomycete fungus that colonises many plant roots, is employed most often to promote the growth of plants (Burragoni and Jeon, 2021). Biopesticides and biofertilizers are becoming formulated with *Trichoderma species* like *T. hamatum, T. harzianum, T. polysporum*, and *T. virideare* because of their ability to colonise root tissues and interact with the host plant via molecular crosstalk, thereby improving nutrient and water uptake, inducing disease resistance, degrading toxic compounds, and ultimately promoting plant growth (Topolovec-Pintarić, 2019). Endophytes defend plants from environmental stresses such as salinity, drought, and others through a variety of mechanisms. One of these mechanisms is the increased production of abscisic acid (ABA), which in turn produces proteins that assist plants in reducing the amount of water lost through transpiration and oxidative stress (Sah et al., 2016). In addition, increased tryptophan production leads to the production of IAA, which is the plant growth hormone auxin and promotes plant growth and rooting (Khan et al., 2019). In a similar fashion, 1-Aminocyclopropane-1-carboxylase (ACC) deaminase hydrolyses the ACC and reduces the production of ethylene, which is responsible for the senescence of the plant, while promoting the production of ammonia and alpha ketobutyrate, which are potential plant growth promoters (Nascimento et al., 2018 **).** Endophytes confer drought tolerance on their hosts by increasing tissue solute accumulation, decreasing water conduction through the leaf, lowering transpiration rates, or thickening the cuticle of the leaf and through osmoregulation for instance, *endophytic Neotyphodium* spp. improves grass plant drought tolerance (Chhipa and Deshmukh, 2019). Secretion of antioxidant metabolites such as ascorbate and glutathione by endophytes reduces host tissue reactive oxygen species and promotes salt stress tolerance (He et al., 2017). These mechanisms work together to improve plant growth under abiotic and biotic stress conditions by increasing root length and density, increasing nutrient supply to plants, suppressing phytopathogens (**Supplementary Table 1**), and improving relative water content, osmotic adjustment, and antioxidant property (Khan et al., 2019). Investigators have shown that fungal endophytes have an important role in the host plant, especially in Phyto-stimulation, phytoremediation, phyto-immobilization, phytotransformation and biological control. In addition to this such fungal endophytes produce secondary metabolites which play a role in the reduction of heavy metal toxicity (Radziemska et al., 2021), (Anamika et al., 2018). These boost the plant’s antioxidative mechanism, leading to detoxification and allowing it to grow in polluted soil, thereby increasing the plant’s resistance to heavy metals (Radziemska et al., 2021). Besides this, fungal endophytes have also several beneficial effect on the host plant which is shown below in **Figure 4**. Hydrophobic, organic molecules with a low molecular weight (300 Da) and a high vapour pressure (0.01 kPa at 20°C) are known as volatile organic compounds (VOCs). Most of these chemicals are derivatives of amino acids, benzenoid compounds, fatty acids, phenylpropanoids, or terpenoids (Kaddes et al., 2019). Endophytic bacteria and fungi produce volatile organic compounds (VOCs) that effectively prevent plant diseases caused by phytopathogens (Kaddes et al., 2019), (Etminani and Harighi, 2018; Etminani et al., 2022).

The endophytic fungus *Trichoderma harzianum*, isolated from the tomato plant *Solanum lycopersicum*, produces the volatile organic compound diterpene. This compound inhibits the growth of the phytopathogen *Botrytis cinerea* by inducing the expression of tomato defence genes related to salicylic acid (SA) (Faucon et al., 2017). Grapevine endophytic bacteria such as *Pantoea* sp. *Sa14*, *Pseudomonas* sp. *Sn48, Pseudomonas* sp. *Ou22*, *Pseudomonas* sp. *Ba35*, *Serratia* sp. *Ba10*, and *Enterobacter* sp. *Ou80* all produce volatile organic compounds (VOCs) that impede the growth of *Agrobacterium tumefaciens* in a number of ways, such as inhibiting the chemotaxis, motility, biofilm growth, and root attachment (Etminani and Harighi, 2018; Etminani et al., 2022) Antiherbivore defences in grasses are bolstered by the presence of the endophytic fungus Epichloe, both through alkaloid-dependent and-independent pathways (Bastias et al., 2017). Epichloe endophytes not only defend host plants against herbivores but also from several pathogens. For instance, the presence of an Epichloe endophyte within plants reduced the symptoms of plant diseases caused by the biotrophic fungal infections *Blumeria graminis*, *Claviceps purpurea, Ustilago bullata, and Laetisaria fuciformis* (Kou et al., 2021).

## 6 Endophytes of industrial significance

Bacterial and fungal endophytes are the greatest sources of enzyme production which can be used in several industries (Patel et al., 2017), (Hirata et al., 2018). Endophyte species produce several enzymes, including proteases, pectinases, amylases, cellulases, xylanases, laccases, lipases, and others, which are significant in many industrial industries (**Table 2**) (Zaferanloo et al., 2014). Additionally, some enzymes generated by endophytic species play crucial roles in a wide range of industries, including the production of biofuels in the energy sector, which is used as an alternative source of conventional fuel; the development of pigments for the food industry; the manufacturing of enzymes to degrade polyurethane; and many more (Mengistu, 2020 **;** Singh et al., 2023). Endophytes Phomopsis, Cephalosporium, Microsphaeropsis, and Nigrospora were isolated from plants *Taxus chinensis* var. *mairei* Mast, *Cupressus torulosa D. Don, Keteleeria davidiana varchienpeii, Sabina chinensis* cv. *Kaizuca and Keteleeria evelyniana Mast*, are known to synthesize enzymes which aid in the extraction of biofuels (Tiwari et al., 2023). In place of toxic chemicals, the textile industry uses a combination of pectinase with amylase, lipase, cellulase, and hemicellulase to digest cotton and remove sizing agents. Pectinase has been investigated extensively in oil extraction from several sources, such as flaxseed, dates, and olives (Haile and Ayele, 2022). Currently, immobilised lipases are used in a wide range of commercial processes, including the manufacture of biosensors, biodiesel and cleansers as well as the organic synthesis of various substances, including cosmetics, meals, medications, fragrances, and tastes (Ismail and Baek, 2020). Xylanase is used as a pre-bleaching agent in the paper and pulp industries. It also has biotechnological applications in the biofuel, food, textile, and feed industries (Singh et al., 2019). Chemical, beverage, textile, food, biofuel, and paper sectors are just a few of the many that rely on the starch-digesting enzyme amylase. It is widely used in the pharmaceutical industry to hydrolyse starch to create various sugars including glucose and maltose, which have a variety of applications. The starch industry uses amylases most frequently to hydrolyze starch during the starch liquefaction process, which turns starch into fructose and glucose syrups (Mehta and Satyanarayana, 2016). Proteases are essential industrial enzymes with numerous uses in chemical and biological reactions. Proteases are also utilised in many other industries, including the production of detergents, the food industry, the tanning of leather, the manufacturing of paper, the recovery of silver from photographic films, the manufacturing of paper, bioremediation procedures and employed therapeutically to cure inflammation and dangerous lesions (Abdel Wahab and Ahmed, 2018; Othman et al., 2018). Cellulases are in great demand across many different industries, including the food and beverage industry, the paper and pulp businesses the manufacturing of textiles, the pharmaceutical field, the cleaning products industry, and the biofuels sector (Raghav et al., 2022). Cellulases are crucial in the selective processing of lignocellulosic biological materials (Payne et al., 2015; Jayasekara and Ratnayake, 2019). Lipopeptides are an important class of secondary metabolites produced by bacterial endophytes and consist of cyclic or linear peptides that are connected to lipophilic molecules. Antibiotic efficacy against numerous diseases places these lipopeptides among the most potent available compounds (Narayanan and Glick, 2022), (al Ayed et al., 2022). Endophytes producing lipopeptides were reported in the medicinal plant *Cordia dichotoma L.*, which is native to the Jammu region. These endophytes belonged to the genera Acidomonas, Alcaligenes, Bacillus, Pseudomonas, Peaenibacillus, Ralstonia, Streptococcus, Micrococcus, and Staphylococcus. Many of the lipopeptide-producing endophytes demonstrated antibacterial activity against a wide variety of bacteria, including *Salmonella typhi, Escherichia coli, Pseudomonas aeruginosa, Bacillus subtilis, Staphylococcus aureus, Klebsiella pneumoniae* (Sharma and Mallubhotla, 2022). *Aspergillus* sp. *A9, Aspergillus* sp. *A36, Penicillium* sp. *P5*, and *Penicillium* sp. *P15*, an endophytic fungus isolated from *M. guianensis*, was found to be an excellent producer of hydrolase enzymes. The lipase and protease produced by Penicillium P15 and *Penicillium* sp. *P5* were able to break down the S. aureus biofilm (Matias et al., 2021). Hydrolytic enzymes like peptidase, amylase, xylanase, and carboxylase are produced by endophytes, which lyse the rigid peptidoglycan or murein that protects bacterial cell walls (Muthu Narayanan et al., 2022). Endophytic hydrolytic enzymes have the ability to degrade the chitin cell walls that are present in pathogenic fungi, which in turn protects plants from becoming infected (Loc et al., 2020).

**Table 2:** Industrial important endophytes and their sources (host).

Host Plant	Endophytes	Functionalities	References
*Coffea Arabica L.*	*Paenibacillus amylolyticus*	Pectinase	(Keggi and Doran-Peterson, 2019)
*C. oblong-folius*	*F. oxysporum PTM7*	Lipase	(Tuangporn, 2012)
*Tithonia diversifolia*	*C. kikuchii*	Lipase	(Costa-Silva et al., 2021)
*Clerodendrum viscosum L.*	*Phoma* sp.	Bio-pigment	(Srivastava et al., 2021)
*Eucryphia cordifolia Cav.*	*Gliocladium roseum*	Myco-diesel	(Strobel et al., 2004)
*Ecuadorian Amazonian plant*	*Pestalotiopsis microspora E2712A*	Polyurethanase	(Russell et al., 2011)
*Zea mays*	*Acremonio zeae*	Xylanase	(Bischoff et al., 2009)
*M. peregrina*	*A. terreus*	Xylanase	(Wu et al., 2022)
*Catharanthus roseus*	Colletotrichum sp., *Fusarium solani, Macrophomina phaseolina, Nigrospora sphaerica*	Amylase, cellulase, protease	(Ayob and Simarani, 2016)
*E. longifolia*	Preussia minima, Alternaria sp.	Amylase	(Zaferanloo et al., 2014)
*A. altissima*	F. proliferatum	Amylase	(Oukala et al., 2021).
*Alpina calcarata (Haw) Roscoe*	Cylindrocephalum sp.	Amylase	(Arawwawala et al., 2012)
*Sporocarp*	*Amanita muscaria, Boletus luridus, Hydnum rufescens, Lactariusa cerrimus, Piceirhiza bicolorata, Piloderma byssinum, P. fallax, Russulachloroides, Suillusluteus luteus*	Protease	(Devi et al., 2020)
*Saraca asoca*	Acremonium sp.	Protease	(Salvi et al., 2022)
*D. hemprichi*	*Penicillium* sp. *Morsy1*	Protease	(El-Gendy, 2010)
*Eucalyptus*	*Hormonema* sp.*, Neofusicoccum luteum, N. australe, Ulocladium* sp.	Laccase	(Fillat et al., 2016)
*Cajanus cajan L.*	*M. verrucaria*	Laccase	(Sun et al., 2017)
*Espeletia* spp.	*P. glabrum*	Cellulase	(Cabezas et al., 2012)
*Centella asiatica*	*Penicillium* sp.	Cellulase	(Devi et al., 2012)
*L. corticata*	*Strain Tahrir-25*	Cellulase	(El-Bondkly and El-Gendy, 2012)
*Opuntia ficus-indica Mill. (Cactaceae)*	*Nigrosporasphaerica, Penicillium aurantiogriseum, Pestalotiopsis guepinii, Xylaria* sp. *1, Acrimonium terrícola, C. cladosporioides, Fusarium lateritium*	Cellulase, Protease, Xylanase	(Bezerra et al., 2012)
*Opuntia ficus-indica Mill. (Cactaceae)*	*Cladosporiums phaerospermum, Phoma tropica, Phomopsisarcheri, Tetraploa aristata, Xylaria* sp. *2*	Protease, Xylanase	(Bezerra et al., 2012)
*Opuntia ficus-indica Mill. (Cactaceae)*	*Aspergillus japonicus*	Cellulase, Pectinase, Protease, Xylanase	(Bezerra et al., 2012)
*Cymbopogon citratus, Murraya koenigii*	*Colletrotrichum, Fusarium, Phoma, Penicillium*	Asparaginase	(Chow and Ting, 2015)
*Asclepias sinaica*	*Alternaria alternate, Penicillium chrysogenum*	Amylase, Cellulase	(Fouda et al., 2019)
*Drimys winteri*	*Bjerkandera* sp.	Cellulase, Phenoloxidase	(Corrêa et al., 2014)
*Glycine max (L.) Merril*	*Rhizoctonia* sp.*, Fusarium verticillioides*	Phytase	(Jain et al., 2014)
*Osbeckia stellata, Camellia caduca, Schima khasiana*	*Mortierella hyaline, Penicillium* sp.	Cellulase, Lipase, Protease, Xylanase	(Bhagobaty and Joshi, 2011a; Bhagobaty and Joshi, 2011b)
*Osbeckia chinensis*	*Paecilomyces variabilis*	Amylase, Lipase, Protease, Xylanase	(Corrêa et al., 2014)
From several plants	*Beauveria bassiana*	Chitinases, lipases and proteases	(Amobonye et al., 2020)

## 7 Role of endophytes in bio-nanotechnology

**Figure 5:**
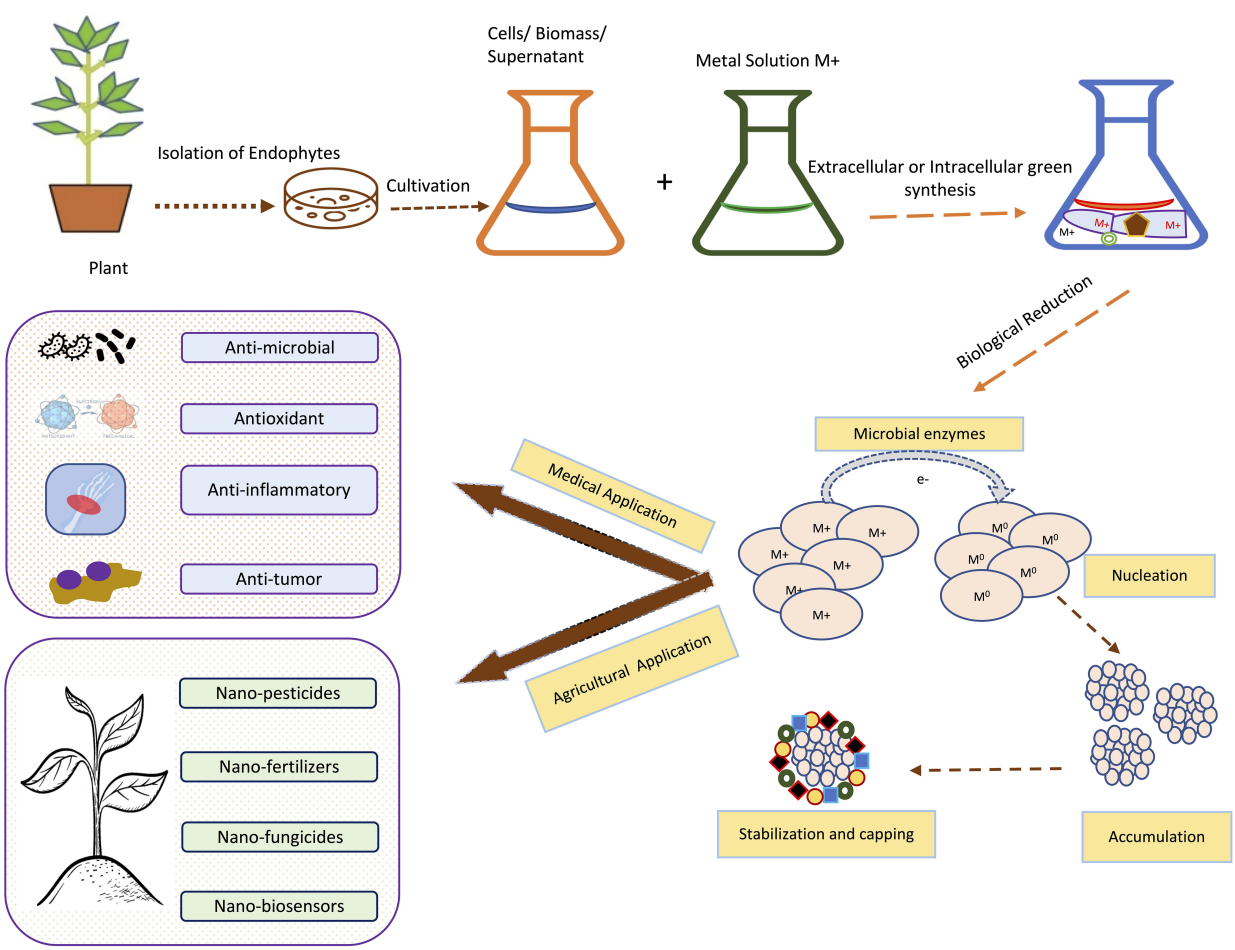
Schematic representation of the general steps for green synthesis of metal-based NPs using endophytic microorganisms isolated from tropical plant adopted from Bogas et al., (Bogas et al., 2022).

Nanotechnology and nanoparticles (NPs) have gained huge attention in the last decade due to their unique and remarkable properties like high surface area to volume ratio and high surface energies (Yadav et al., 2020). Due to these features, NPs are widely used in medicine, research, drug delivery, electronics and environmental clean-up (Modi et al., 2022). When it comes to drug delivery and medicine biocompatible and non-toxic nanomaterials are the first preference. So, biocompatible NPs could be easily synthesized by using fungal and bacterial endophytes. From the various pieces of literature, it has been revealed that numerous investigators have used both prokaryotic and eukaryotic endophytes for the synthesis of both metal NPs and metal oxide NPs (Ahmad and Kalra, 2020; Modi et al., 2022). All these methods mainly involve bottom-up approaches which involve exposure of metallic ions to the desired endophytes under desired conditions. Investigators have proven that the positively charged metal ions come closer to the negatively charged endophytic surfaces by electrostatic attraction. Further, these ions then get transported to the internal structure of the endophytes via ions channels where the metal ions get reduced to their zero-valent atomic states., which further then get aggregated to form NPs (Yadav et al., 2020), (Dhara et al., 2023). **Figure 5** is showing basic steps involved in the green synthesis of NPs by using endophytic microorganisms.

**Figure 6:**
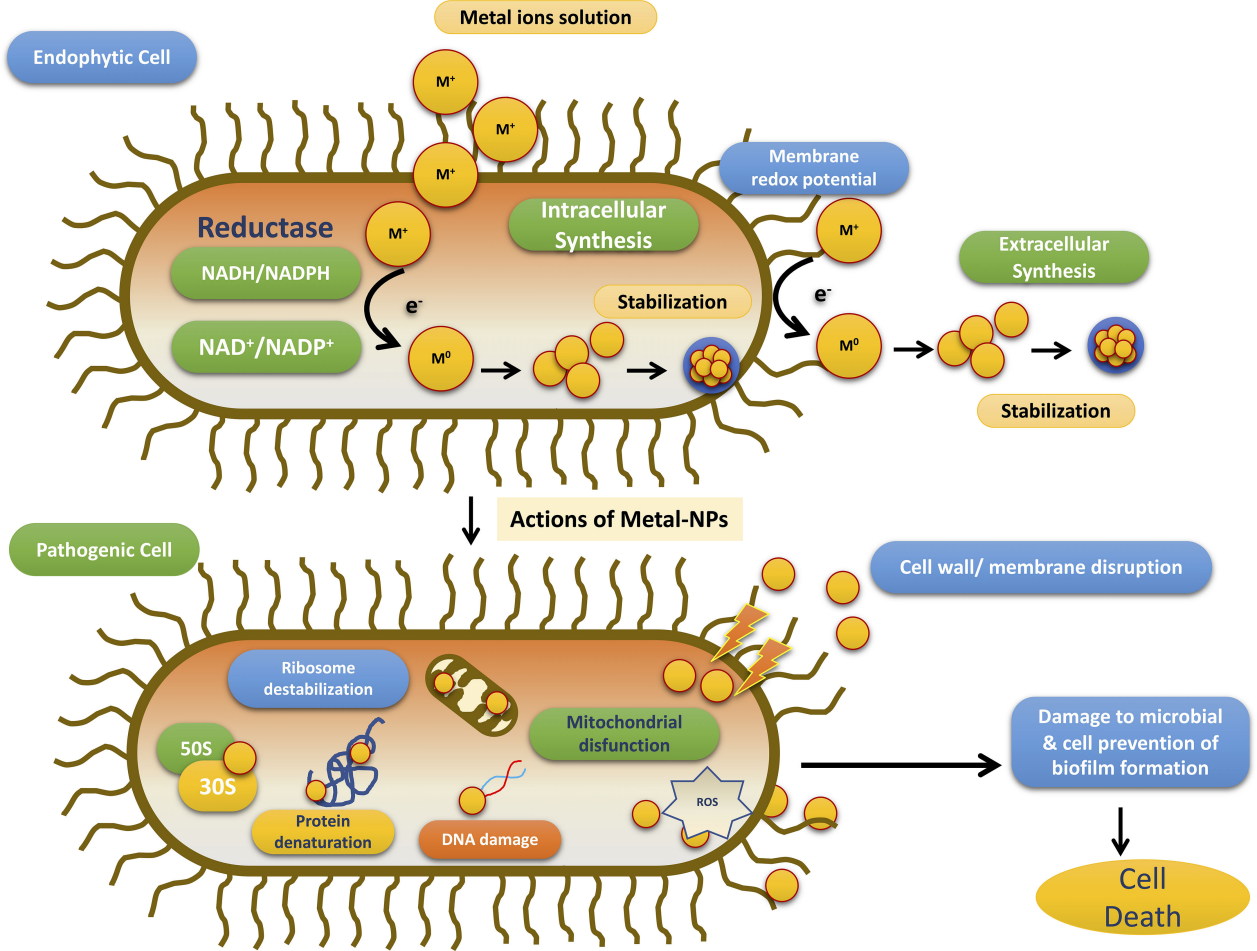
Schematic diagram for the steps involved in the antimicrobial activity of NPs synthesized by endophytes adopted from Bogas et al., (Bogas et al., 2022).

**Figure 7:**
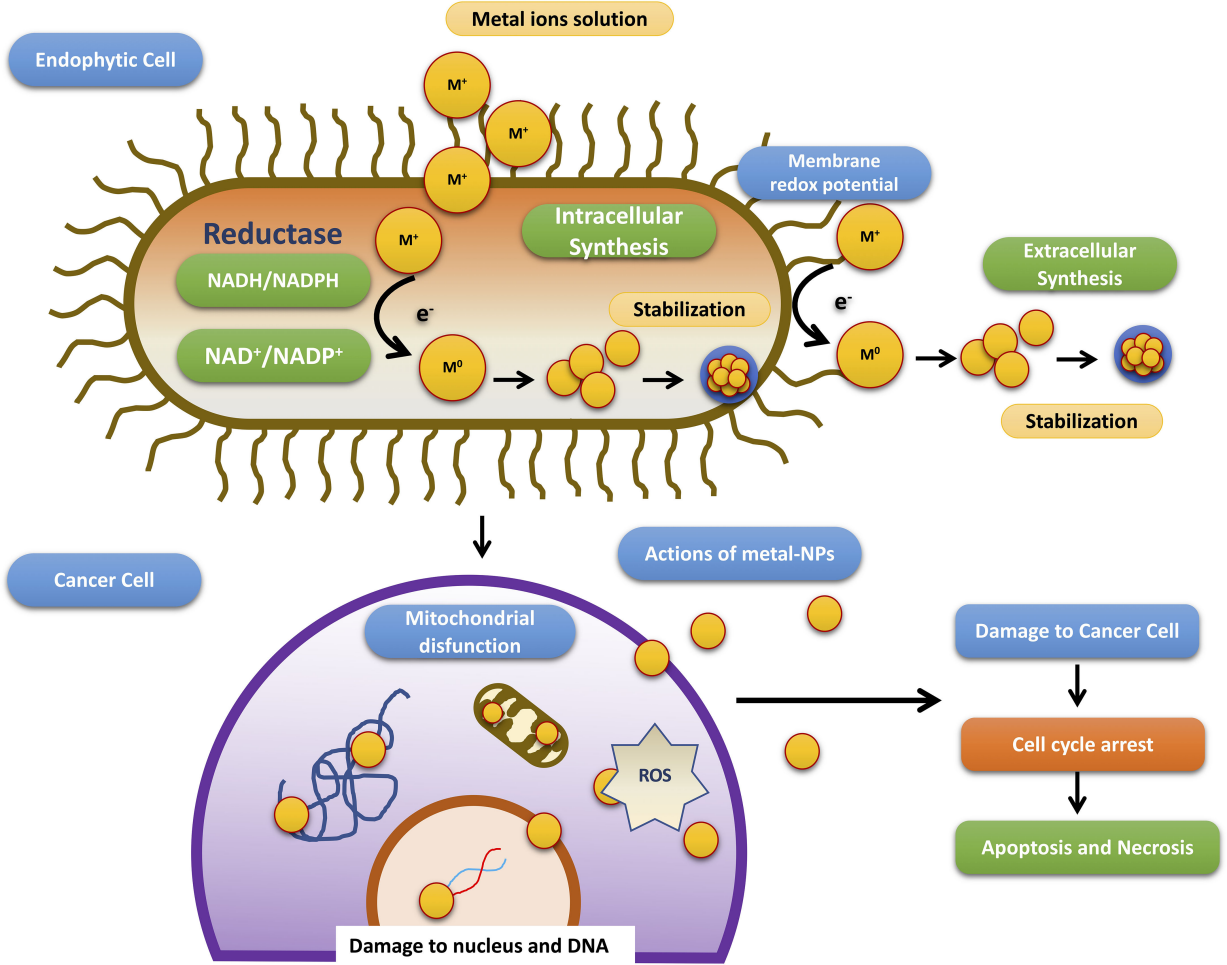
Schematic diagram representing steps involved in the anti-cancer activity of NPs synthesized by endophytes adopted from Bogas et al., (Bogas et al., 2022).

Till date investigators have synthesized gold, silver, and copper metal NPs from the endophytes, which have been used in all the domains of science. In addition to this, investigators have reported the synthesis of zinc sulfide, copper oxide, cobalt oxide, nickel oxide, etc from endophytes isolated from terrestrial and marine regions. Fadiji and their colleagues showed the role of various NPs synthesized from the bacterial and fungal endophytes in sustainable agriculture by enhancing plant growth and improving disease resistance (Fadiji et al., 2022). Bogas and their team have shown that these endophytes could act as biofactories for nanoparticles. NPs synthesized from such endophytes have immense potential in healthcare applications (Bogas et al., 2022). In addition, Rathore and his colleagues have placed an emphasis on bacterial endophytes, discussing the recent biomedical scope of these organisms, as well as their synthesis, associated challenges, and importance in bio-nanotechnology (Rathore et al., 2022). Mishra and their groups have also emphasized the green synthesis of NPs by using fungal endophytes which have easy scale-up, downstream processing and eco-friendly nature (Misra et al., 2021). The majority of these NPs synthesized from the endophytes have a role as an antimicrobial agent or as an anti-cancer agent. The antimicrobial activity of endophyte-mediated synthesis NPs is shown in **Figure 6** while **Figure 7** is showing anticancer activity of the NPs synthesized from endophytes. **Table 3** is showing a summarized form of various nanoparticle syntheses from endophytes, along with their applications.

**Table 3:** Nanoparticles synthesis from endophytes, sources and their applications.

Nanoparticles	Plant & their parts	Endophytes	Applications	References
AgNPs (Silver nanoparticles)	Roots of tropical plants	Bacteria SYSU 333150, Isoptericola sp.*, Streptomyces laurentii*	ABA, ACA	(Eid et al., 2020)
	*Borszczowia aralocaspica Bunge* (roots), *Raphanus sativus* & *Azadirachta indica* (leaves)	Endophytic strain SYSU 333150 Supernatant of fungi *Alternaria sp, Aspergillus* sp.*, Chaetomium* sp.*, Cladosporium* sp.*, Colletotrichum* sp.*, Curvularia* sp.*, Guignardia* sp.*, Penicillium* sp.*, Pestalotia* sp.*, Pestalotiopsis* sp., and *Phomopsis* sp.	ABA against *Staphylococcus warneri*	(Dong et al., 2017)
	*Curcuma longa* (turmeric)	*Penicillium Guignardia mangiferae A. terreus*	AMA against MDR *E. coli and S. aureus*, ACA AMA, against *S. aureus & B. subtilis*	(Singh et al., 2014) (Balakumaran et al., 2015) (Balakumaran et al., 2016)
	*Calotropis procera* (leaves extract)	Supernatant of *Penicillium* sp.*, Alternaria* sp.*, Aspergillus* sp. and *Cladosporium* sp.	Antibacterial activity against *E. coli* and *B. subtilis*	(Chowdhury et al., 2016)
	*Rhizophora magle* and *Laguncularia racemose*	*Aspergillus tubingensis* and *Bionectria ochroleuca*	Antimicrobial activity against *Pseudomonas aeruginosa*	(Rodrigues et al., 2013)
	*Stypandra glauca*	*Aspergillus niger*	Antibacterial activity against *E. coli* and *P. aeruginosa;*	(Hemashekhar et al., 2019) (Verma et al., 2009)
	*Exserohilum rostrata*	Cell-free extracts of *Ocimum tenuiflorum*	ABA, inhibit bacterial biofilm formation of *P. aeruginosa* and *S. aureus*	(Bagur et al., 2020)
	*Tinospora cordifolia*	*Penicillium* sp.	ACA	(Bagur et al., 2022)
	*Bertholletia excelsa* (Brazil nut) seeds	*Trichoderma* spp.	ABA	(Ramos et al., 2020)
AuNPs (Gold nanoparticles)	Enoki mushroom (*Flammulina velutipes*)-fruiting bodies	*A. terreus*	AMA, *against S. auresus & B. subtilis*, Methylene blue dye removal	(Balakumaran et al., 2016)
	*Sargassum wightii (*seaweed*)*	*C. cladosporioides* (marine endophytic fungi)	Antioxidant and AMA	(Manjunath et al., 2017)
	*Commiphora wightii*	*Cladosporium* sp.	ACA	(Munawer et al., 2020)
	*Azadirachta indica*	Cell extracts of *Aspergillus* sp.	Antimicrobial activity against *C. albicans, P. fluorescens* and *E. coli.*	(Hemashekhar et al., 2019), (Verma et al., 2009)
	*Rauvolfia tetraphylla* (roots)	Cell-free extracts of *Alternaria* sp.	Antibacterial activity against *E. coli, P. aeruginosa, K. pneumonia* and *S. aureusas* well as antioxidant and antimitotic activities	(Hemashekhar et al., 2019)
CuO NPs	*Aegle marmelosa* & *Origanum majorana*	*Aspergillus terreus* (biomass and supernatant)	ACA	(Mani et al., 2021)
	*Calendula arvensis* (leaves)	*Streptomyces capillispiralis* Ca-1 (marine actinomycetes), *Phaeoacremonium* sp.	AMA, biocontrol of plant pathogens	(Hassan et al., 2018)
ZnS quantum dots	*Nothapodytes foetida* (leaves)	*Aspergillus flavus*	Environmental and biomedical application	(Uddandarao and Mohan, 2016)
ZnS: Gd NPs	*N. foetida* (leaves)	*Aspergillus flavus*	Sensing; Fluorescence-Based Metal Detection	(Uddandarao et al., 2019)
Co_3_O_4_ NPs	*Morus nigra*	*Asp terreus*	AO and AMA	(Mousa et al., 2021)
Fe_3_O_4_	*Origanum majorana*	*Asp terreus*	AO and AMA	(Mousa et al., 2021)
NiO NPs	*Origanum majorana*	*Asp terreus*	AO and AMA	(Mousa et al., 2021)
CoO NPs	*N. foetida* (leaves)	*Aspergillus nidulans*	Electrical applications	(Vijayanandan and Balakrishnan, 2018)
Micro and nano TiO_2_	Roots of Sorghum bicolor	*Trichoderma citrinoviride*	ABA against *Pseudomonas aeruginosa*	(Arya et al., 2021)

## 8 Conclusion

Endophytes are bacterial and fungal species beneficial to plants by fulfilling their requirements for growth and protection. Recent applications of endophytes in the agriculture sector not only accelerate plant growth by providing tolerance against various stresses but also reduces the use of numerous agrochemicals like chemical fertilizers and pesticides and this would make agriculture more sustainable and productive. In addition, the exploration of the insecticidal, antimicrobial, and pest-control activities of endophytes will make them good friends of farmers. Endophytes produce several bioactive compounds with huge industrial and medicinal applications as well as can be involved in the bio-transformation of hazardous chemicals like toxins, pollutants and heavy metals. Although the bio-transforming activities of endophytes is still in their infancy. Thus, future efforts should focus on the industrial and medicinal applications of the bio-transforming endophytes and strengthen their eco-friendly and cost-effective approaches in food safety and in the pharma sector. Endophytes exert various therapeutic activities such as anti-cancer, anti-diabetic, anti-inflammatory etc activities by their bioactive compounds. To date, there is no report on commercially available antibiotics derived from endophytes. Intensive research is required which emphasizes the development of new drugs or antibiotics from endophytes and their mechanism of action. The use of endophytic microbes is a relatively new area of study for the environmentally friendly synthesis of nanoparticles, especially when compared to saprophytic microorganisms. Endophyte-derived NPs have potential applications in medicine, including the elimination of multidrug-resistant bacteria, the transport of genetic elements in genetic engineering, and the detection of disease. At the interface of biology and nanotechnology, this area of study has the potential to usher in a plethora of novel nanomaterials. Incorporating metagenomics, metabolomics, and metabolic profiling approaches for elucidating the biosynthetic pathways adopted by endophytes and plants, as well as creating protein-protein interaction maps, and exploring endophytic nanoparticles, will greatly illuminate future applications of endophytes in agriculture, environment, medicine, and industry.

## Author contributions

NC, ND, AG, RV and B-H J contributed in conceptualisation, supervision, review editing and writing, and VY, MC, UB, RG and RC reviewed the manuscript, involved in formal analysis, validation and review editing. MA, LE, WA and NC prepared the final draft. MA, LE, WA, RV and B-HJ contributed in visualisation, supervision, review editing and funding acquisition and RV, RG, B-HJ finalized and submitted the manuscript. All authors contributed to the article and approved the submitted version.
