# Supplementary material for: Towards further understanding the applications of endophytes: enriched source of bioactive compounds and bio factories for nanoparticles
Source: Front Plant Sci. 2023 Jul 10;14:1193573. doi: 10.3389/fpls.2023.1193573 (PMC10364642; doi:10.3389/fpls.2023.1193573)
Supplement: Supplementary file 1 [file Table_1.docx]

**Table 1** Endophytes with their medicinal properties

| **Host Plant** | | **Endophytes** | **Compounds** | **Properties** | **References** |
| --- | --- | --- | --- | --- | --- |
| *Ligusticum chuanxiong* | | *Bacillus subtilis* | Ligustrazine (Tetramethylpyrazine)  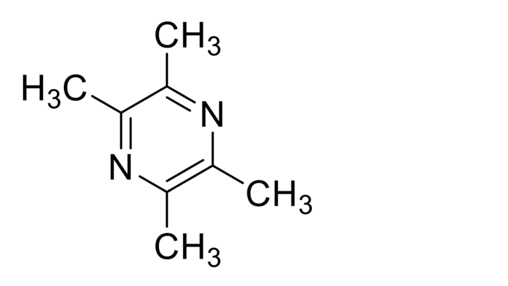 | Treating ischemic vascular-related diseases | (Yin et al., 2019) |
| *Catharanthus roseus* | | *Microbacterium sp.* | Vindoline  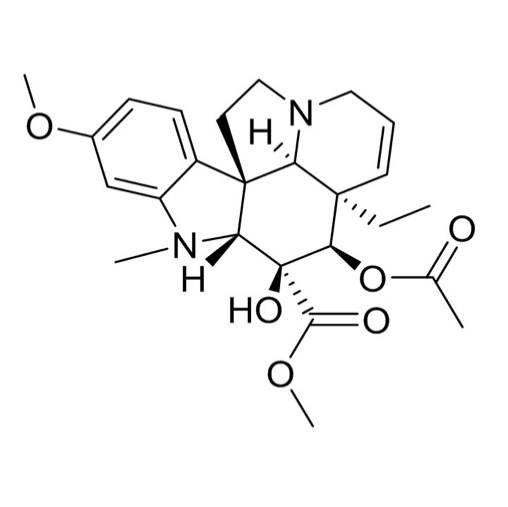 | Treating Hodgkin’s disease and acute leukemia | (Adrees et al., 2019) |
| *Gelsemium elegans* | | *Guignardia mangiferae* | Guignarderemophilanes A-E  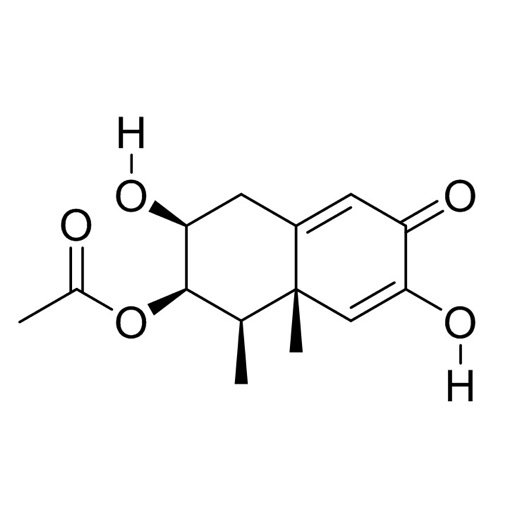 | Anti-inflammatory | (Liu et al., 2015) |
| *Terminalia morobensis* | | *Pestalotiopsis microspora* | Pestacin, isopestacin & 1, 3-dihydro isobenzofurans  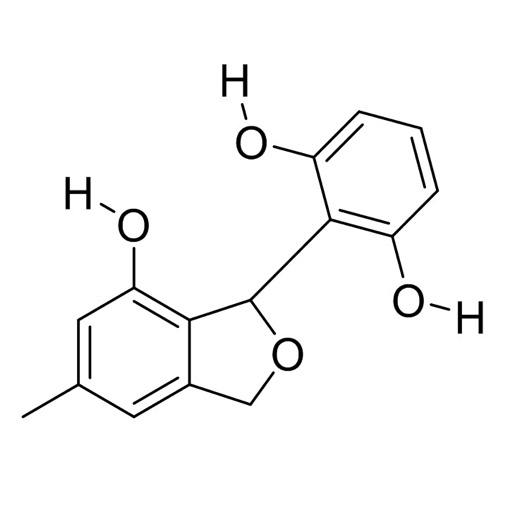 | Antioxidant, antimycotic, anti-hypercholesterolemic, antimalarial | (Toghueo, 2020) |
| *Solanum xanthocarpum* | | *Phomopsis vexans* | Lovastatin  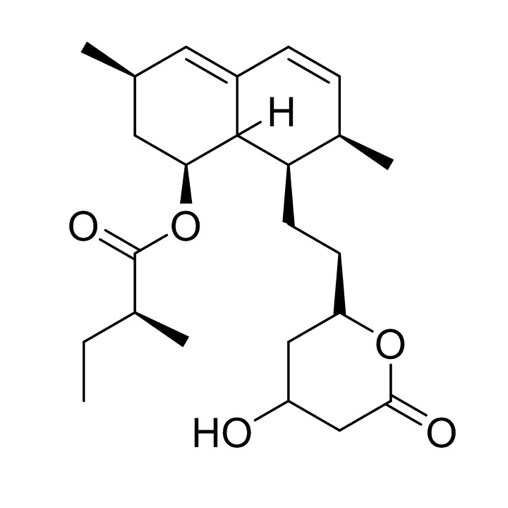 | Lowering blood cholesterol | (Parthasarathy & Sathiyabama, 2015) |
| *Smallanthus sonchifolius & Viguiera arenaria* | | *Curvularia spp. Phomopsis spp.* | Stemphyperylenol, 3,4-dimethyl-2-(4 -hydroxy-3 ,5 -dimethoxyphenyl)-5- methoxy-tetrahydrofuran  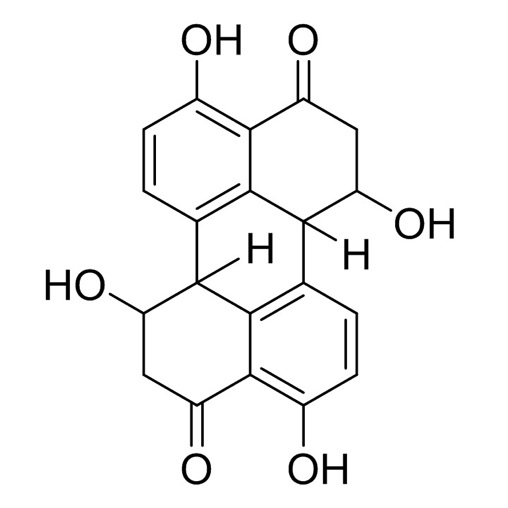 | Antimalaria/antiparasite | (Caruso et al., 2020) |
| *Monstera sp.* | | *Streptomyces sp.* | Coronamycin  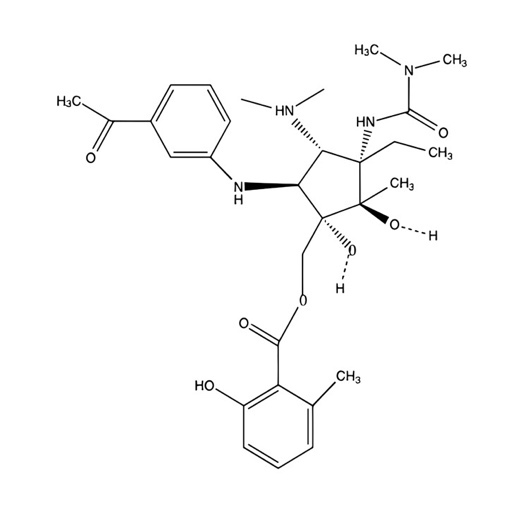 | Antimalarial, antifungal activity (AFA) | (Ezra et al., 2004) |
| *Aegiceras corniculatum (L.) Blanco* | | *Emericella sp.* | Emerimidine A, Emerimidine B, Emeriphenolicins A, Emeriphenolicins D, Aspernidine A, Aspernidine B, Austin, Austinol, Dehydroaustin, and Acetoxydehydroaustin  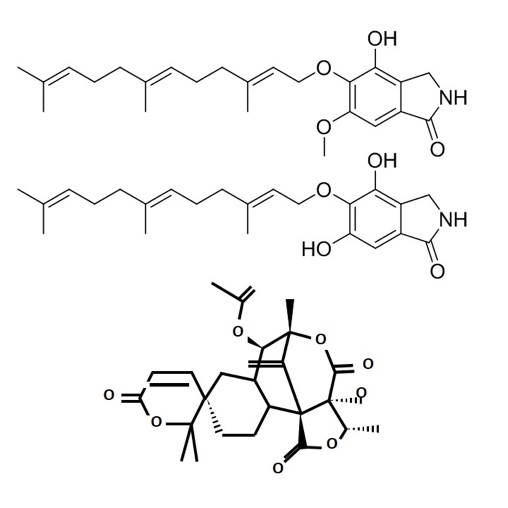  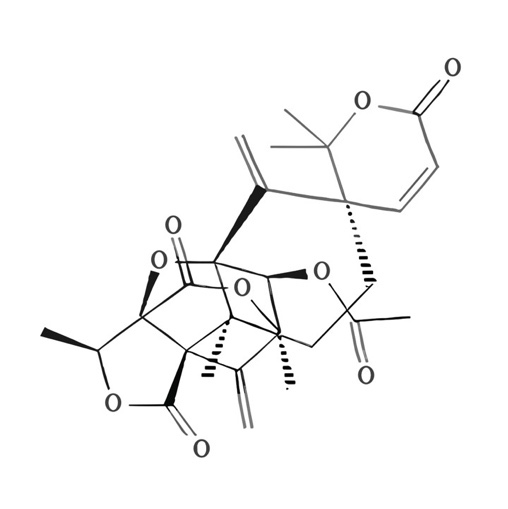 | Antiviral | (G. Zhang et al., 2011) |
| *Quercus sp.* | | *Cytonaema sp.* | Cytonic acids A and D  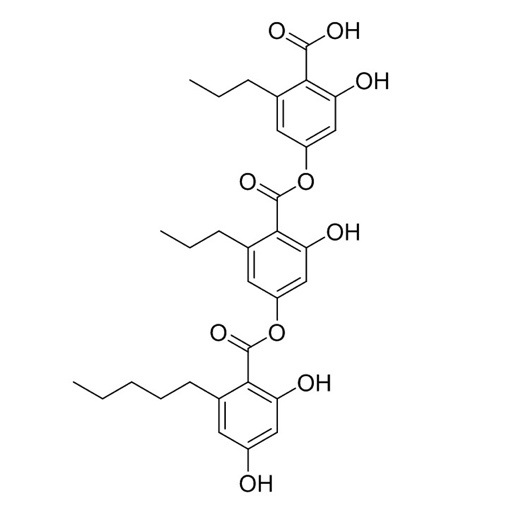 | Antiviral | (Tiwari et al., 2023) |
| *Sabina recurve* | | *Fusarium oxysporum* | Cyclosporine  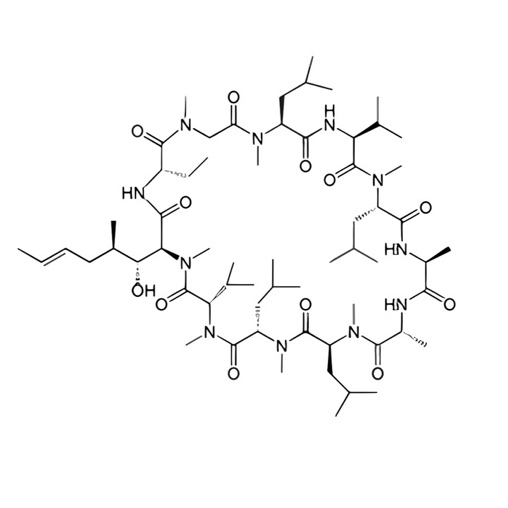 | Antiviral | (Jia et al., 2016) |
| *Kandelia candel (L.) Druce* | | *Guignardia sp*. KcF8 | Guignardin A-F, Palmarumycin C1  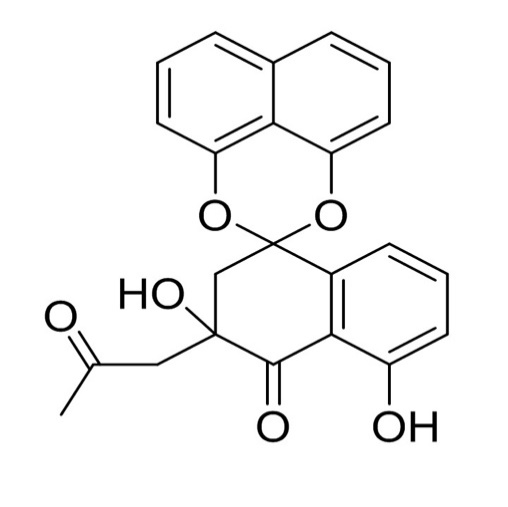  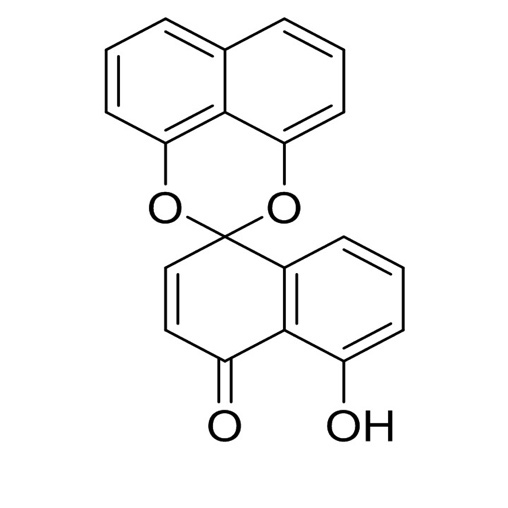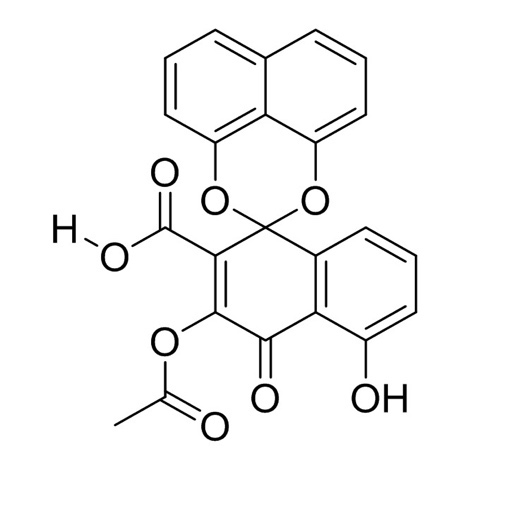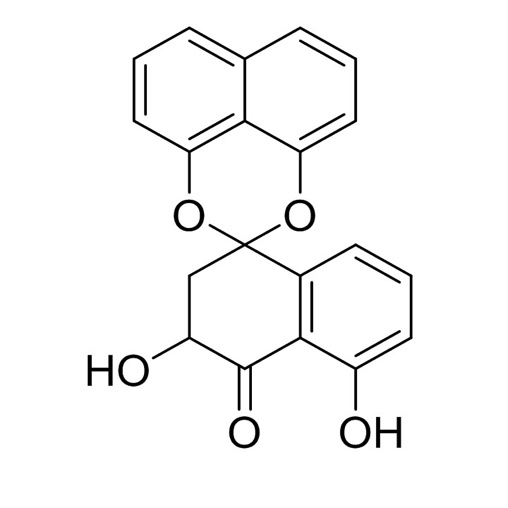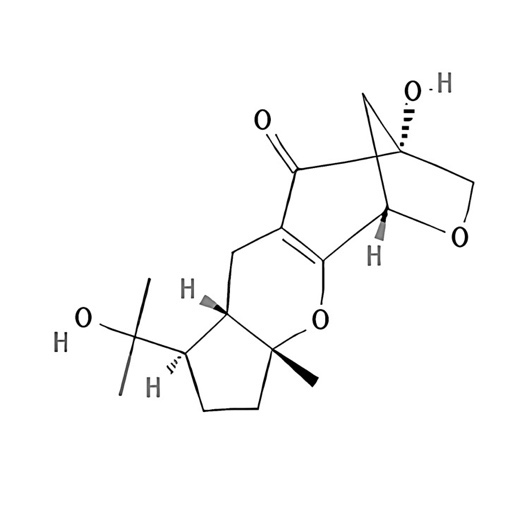 | Antimicrobial activity (AMA), cytotoxic, protein inhibitor | (Ai et al., 2014) |
| *Bruguiera gymnorrhiza* | | *Streptomyces sp* | Xiamycin Trichodones A-C  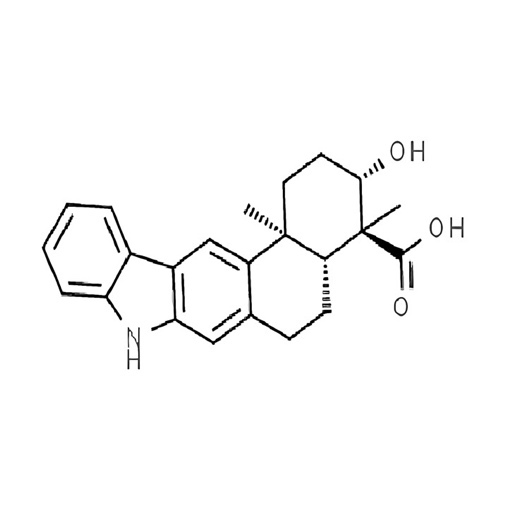 | Anti-HIV | (L. Ding et al., 2010) |
| *Bruguiera gymnorrhiza* | | *Streptomyces sp* | Xiamycin  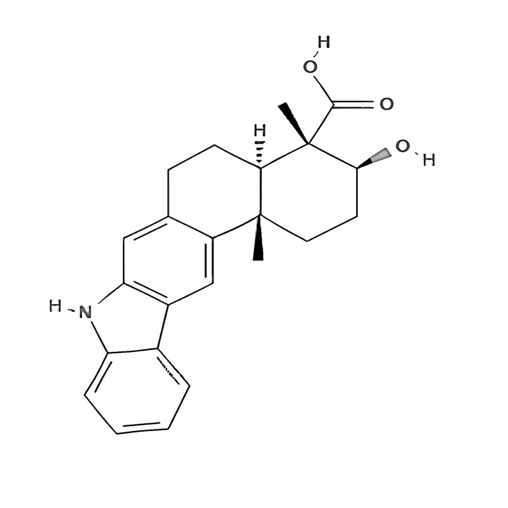 | Anti-HIV | (L. Ding et al., 2010) |
| *Allium tuberosum* | | *Streptomyces sp* | 6-Prenylindole  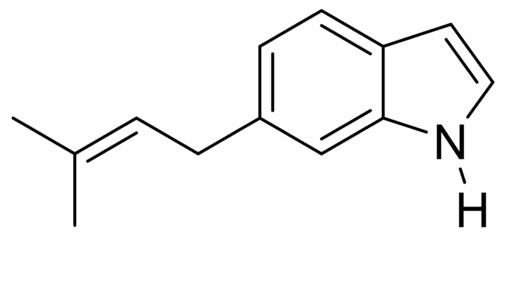 | Antifungal | (R. Singh & Dubey, 2018)  (Huang, 2019) |
| Glycine max | | *Streptomyces sp* | 3-Acetonylidene-7-Prenylindolin-2-1,7-Isoprenylindole-3-carboxylic acid  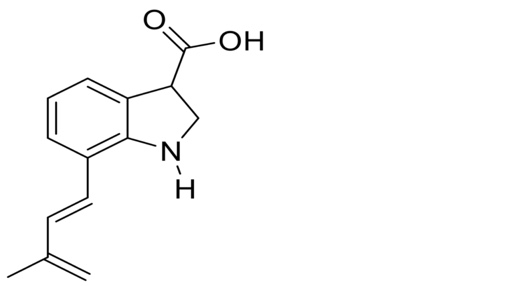 | Antifungal | (H. Yan et al., 2019a; Y. Yan et al., 2014)  (Jain et al., 2014) |
| Ginkgo biloba | | *C. globosum* | Chaetomugilin A & D  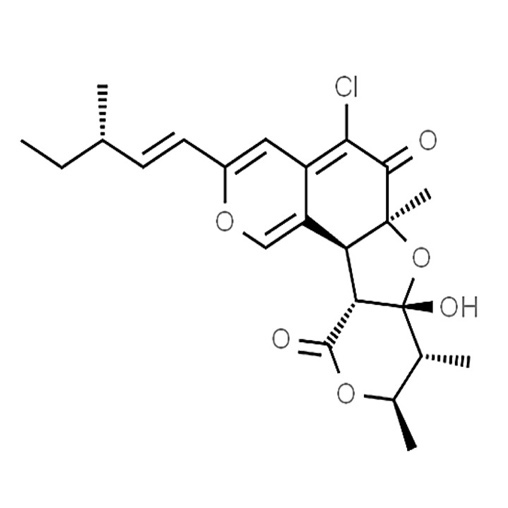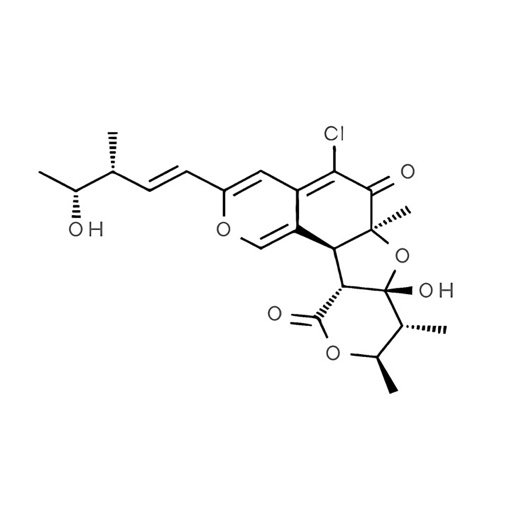 | Antifungal | (Qin et al., 2009) |
| *Rhyncholacis penicillata* | | *Serratia marcescens* | Oocydin A  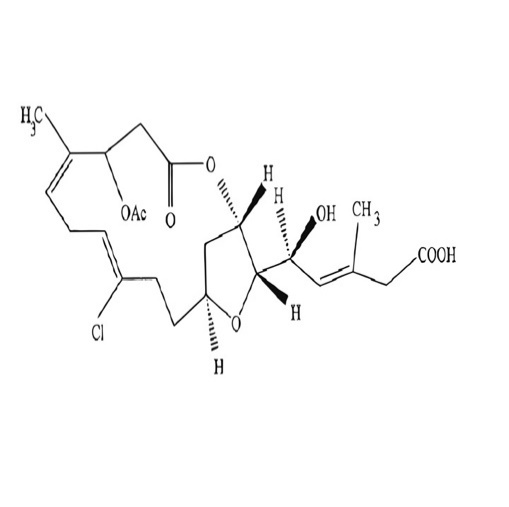 | Antifungal | (Strobel et al., 2004) |
| Oak and various plants | Endophytic fungus sp. | α-Pyrones, and Chromones 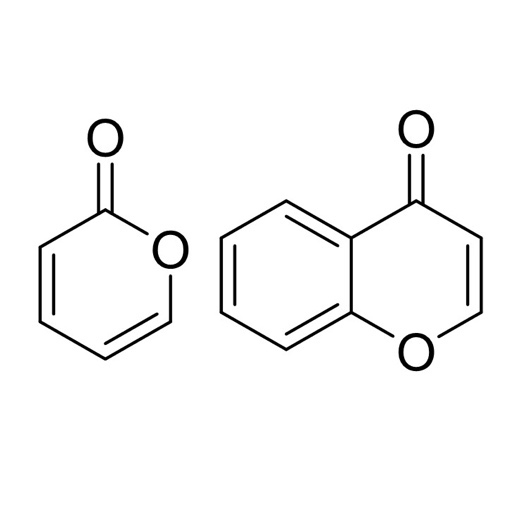 | Antifungal | (Wen et al., 2022) |  |
| Citrus limon | | *Penicillium digitatum,*  *P. citrinum* | Tryptoquialanine A, Tryptoquialanine C, 15-dimethyl-2-epi-fumiquinazoline A, deoxytryptoquialanone, Citrinadin A, Deoxycitrinadin A, Chrysogenamide A  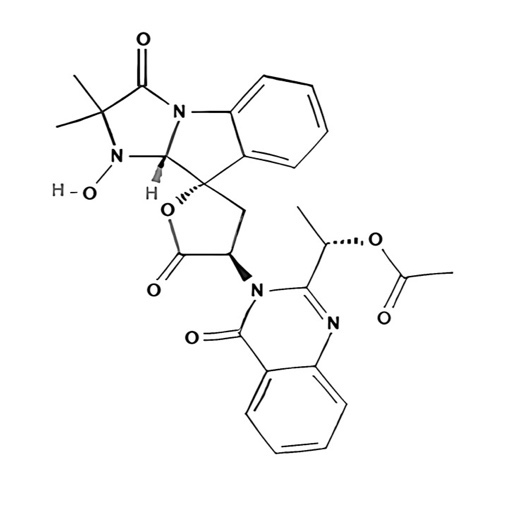  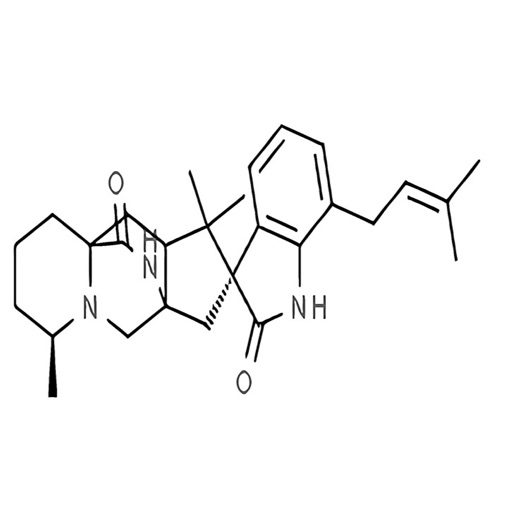  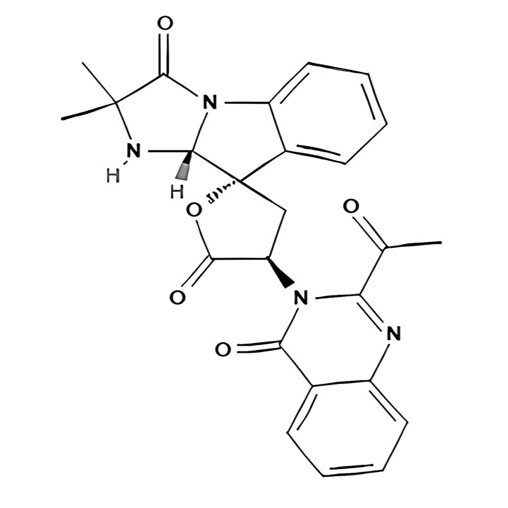 | Antifungal | (J. H. Costa et al., 2019; L. M. Costa et al., 2017) |
| *Excoecaria agallocha L.* | | *Phomopsis sp.* | Phomopsin A, Phomopsin B, Phomopsin C, Cytosporone B, Cytosporone C  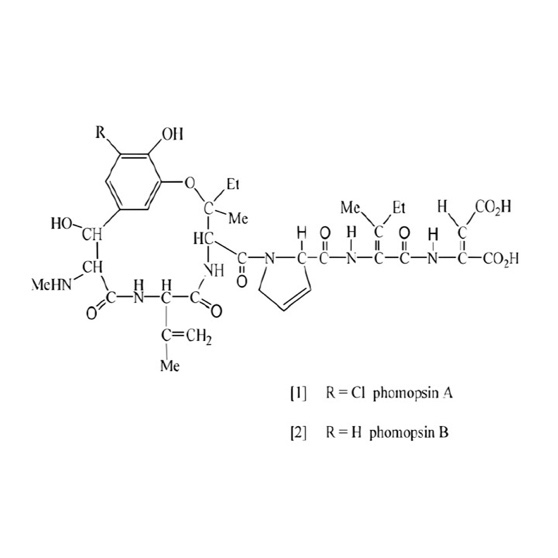  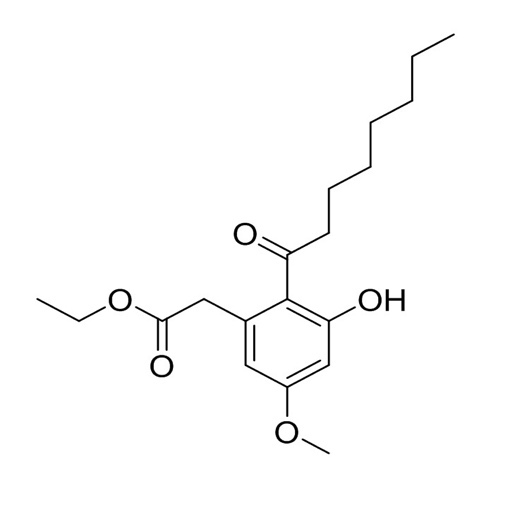  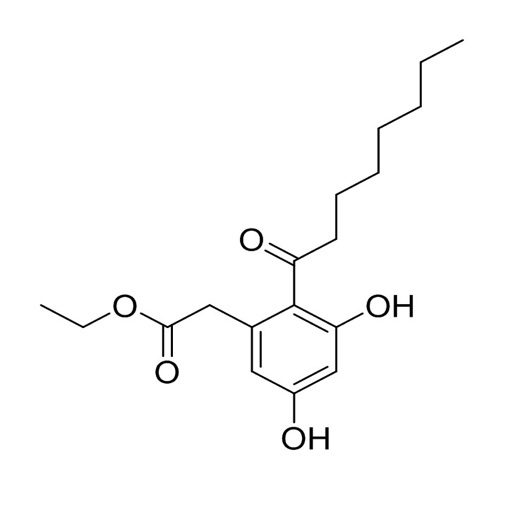  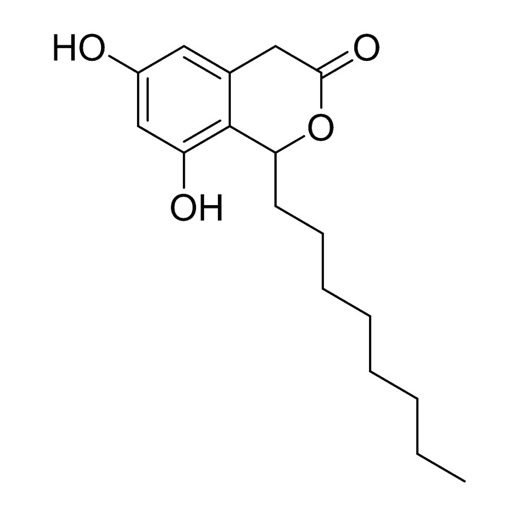 | Antifungal | (W. Ding et al., 2016) |
| *Lychnophora ericoides* | | *Streptomyces sp* | 2,3-dihydro-2,2-dimethyl-4(1H)- quinazolinone  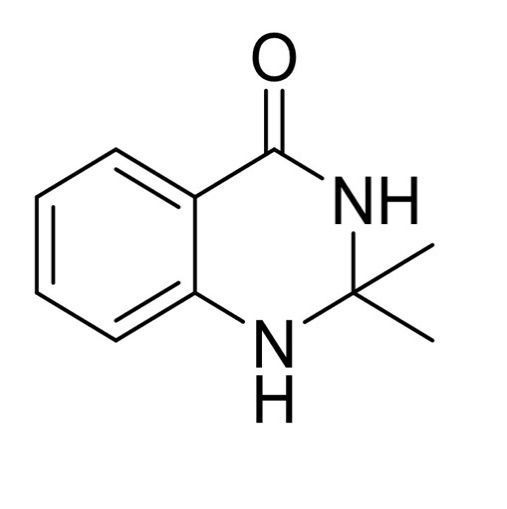 | Anticancer | (Conti et al., 2016) |
| *Ephedra foliata* | | *Paenibacillus polymyxa* | Daunorubicin  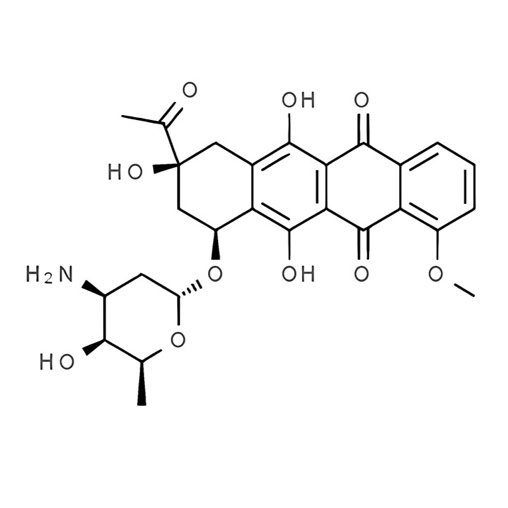 | Anticancer | (Ghiasvand et al., 2020; Peng et al., 2017) |
| *Panax ginseng* | | *Burkholderia sp.* | Ginsenoside Rg3  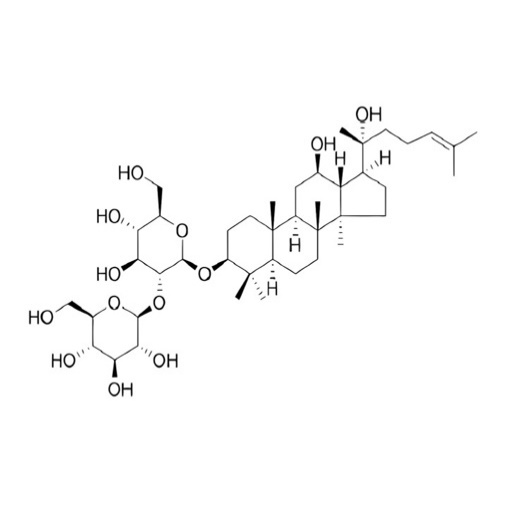 | Anticancer | (Fu et al., 2017) |
| *Panax ginseng* | | *Agrobacterium sp* | Ginsenoside Rh2  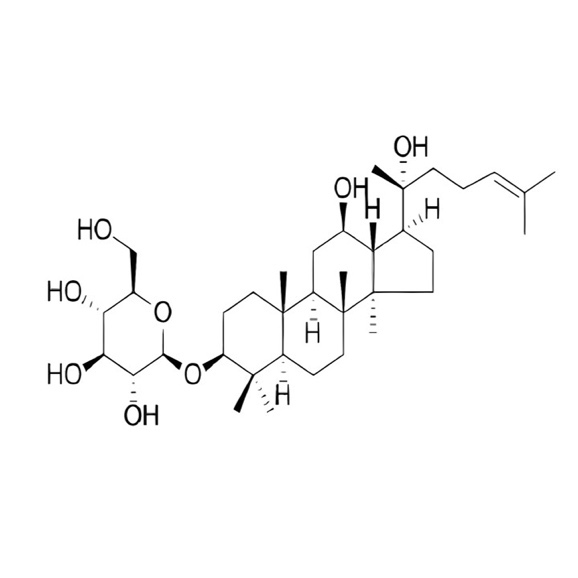 | Anticancer | (H. Yan et al., 2019b) |
| *Taxus celebica (Warb.)* | | *Fusarium solani* | Paclitaxel  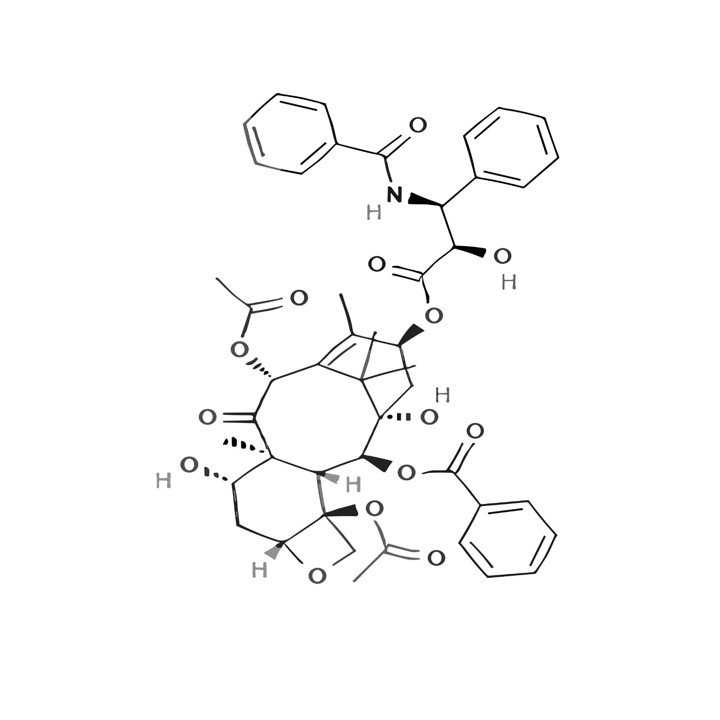 | Anticancer | (Swamy et al., 2022) |
| *Juniperus communis L. Horstmann* | | *Aspergillus fumigatus, Fresenius* | Deoxypodophyllotoxin  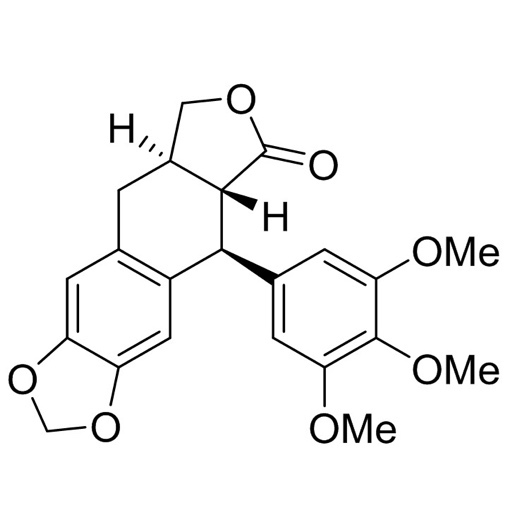 | Anticancer | (Kusari et al., 2009) |
| Mangrove plant | | *Halorosellinia sp., Guignardia sp.* | Anthracenedione derivatives  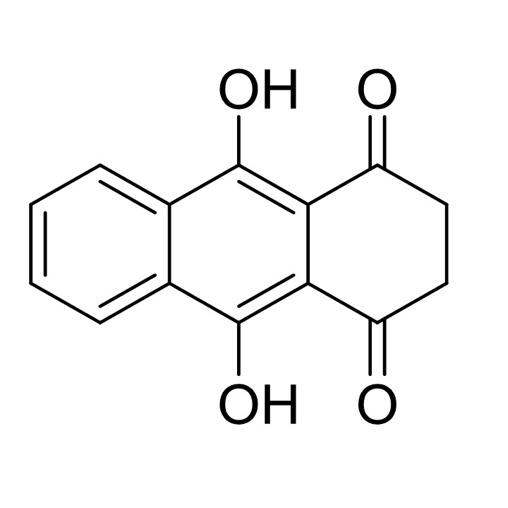 | Anticancer | (J. Y. Zhang et al., 2010) |
| *Maytenus hookeri* | *Putterlickia verrucose, Streptomyces sp, Putterlickia retrospinosa* | Maytansinoids  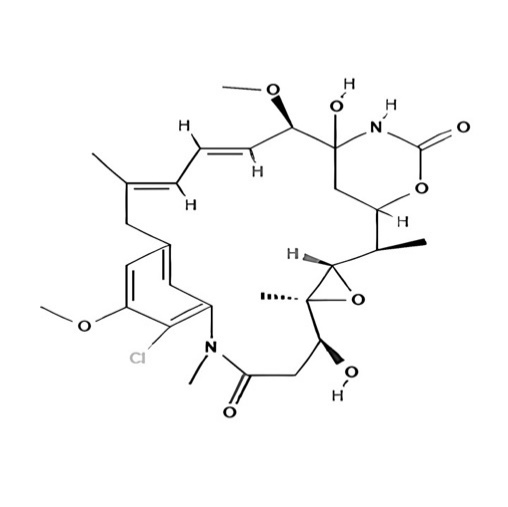 | Anti-cancer | **(al Ayed et al., 2022)** |  |
| *Lychnophora ericoides* | *Streptomyces sp.* | Cyclic Analogs | Anti-cancer | (Conti et al., 2016) |  |
| *Are Vochysia divergens* | | *Aeromicrobium ponti* | 1-Acetyl-β-carboline  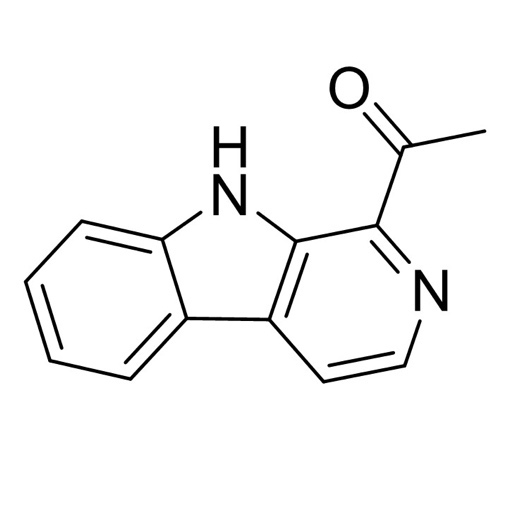 | Antibacterial | (Gos et al., 2017) |
| *Saurauia scaberrinae* Hemsley | | *Phoma sp.* | Usnic acid, Cercosporamid, Phomodione  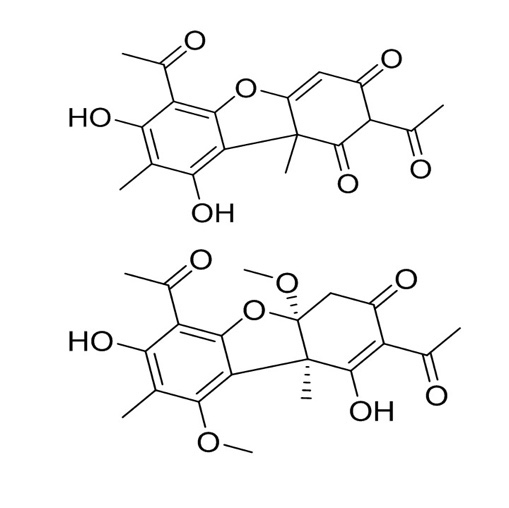 | Antibacterial | (Das et al., 2023; Uzma & Chowdappa, 2021) |
| *Zingiber officinale* | | *Nocardiopsis sp.* | Trans cinnamic acid  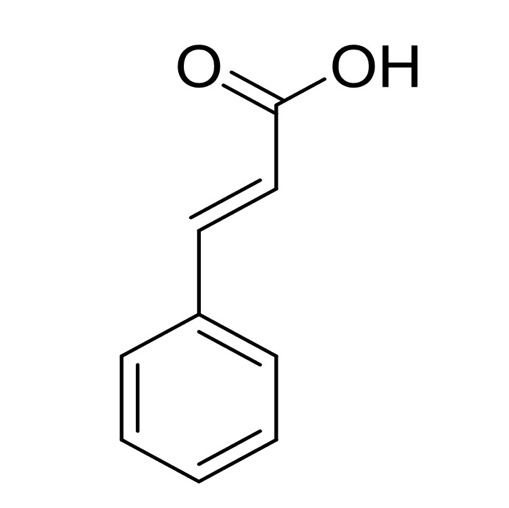 | AMA | (Sabu et al., 2017) |
| *Thymus vulgaris* | | *Bacillus subtilis* | Bis (2-ethylhexyl) phthalate  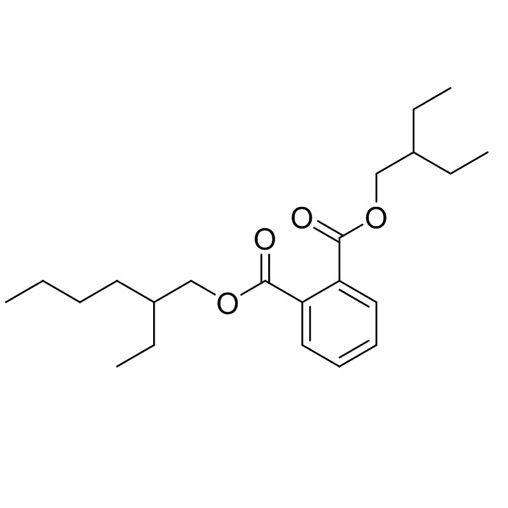 | AMA | (Abdelshafy Mohamad et al., 2020) |
| *Taxus baccata L.* | | *Fusarium solani* | 8-octadecanone, 1-tetradecene, 8-pentadecanone, octylcyclohexane and 10-nonadecanone  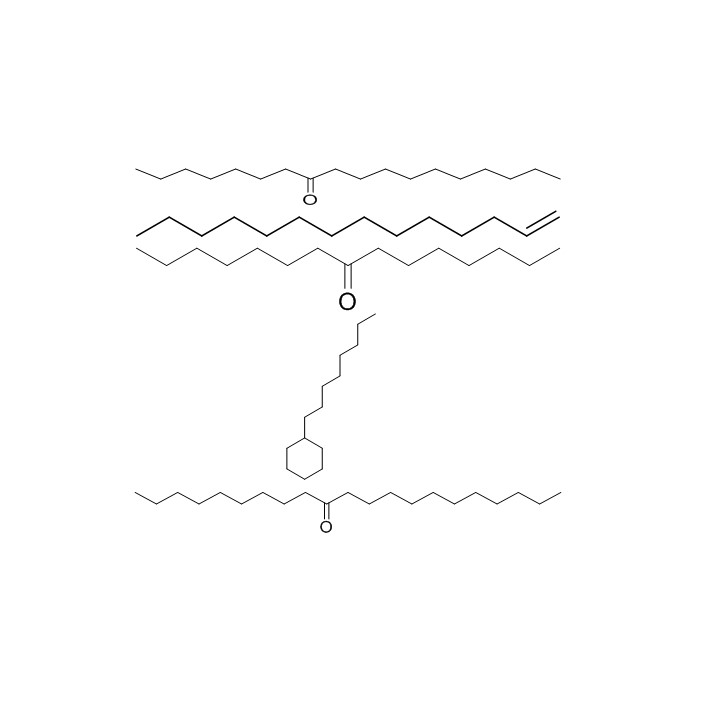 | Antimicrobial activity against *Klebsiella pneumoniae* and *Shigella flexneri* | (Anamika et al., 2018) |
| *Huperzia serrata* | | *Shiraia sp.* | Huperzine A  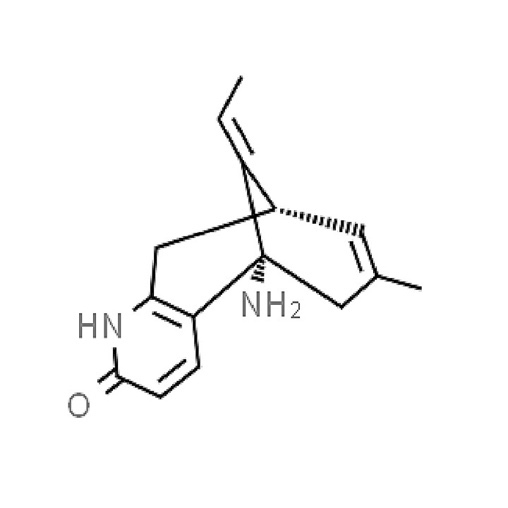 | Potential anti-dementia drug (acetylcholinesterase inhibitor) | (Wang et al., 2011) |
| *G. biloba* | | More than thirty endophytes’ species | Terpenes trilactones:  Ginkgolides (diterpenes) and Bilobalide (sesquiterpene)  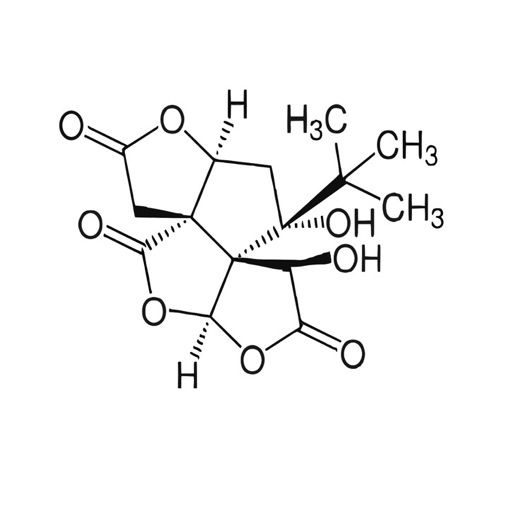  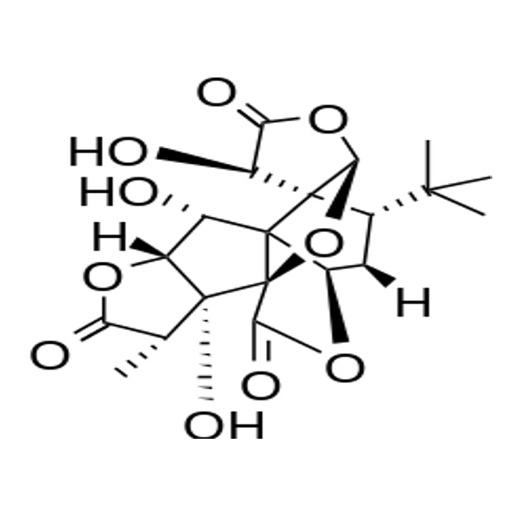 | Neuroprotective effects,  Anti-tumor,  Anti-apoptotic,  Antioxidant, and Anti-inflammatory activities | (Toghueo, 2020) |
| *Vaccinium angustifolium* | | *Xylaria ellisii* | Griseofulvin, Eremophilane ( sesquiterpene); sordarin (pheromones)  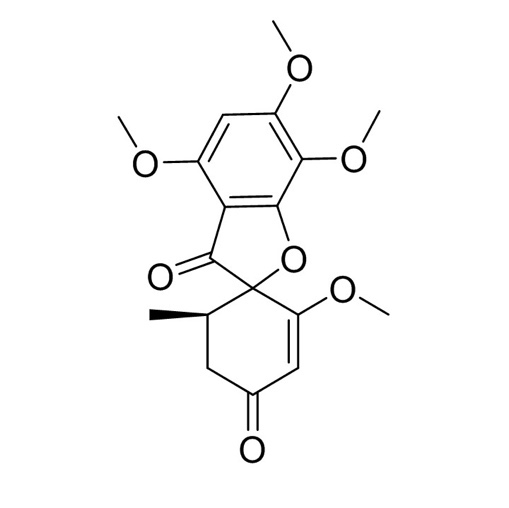  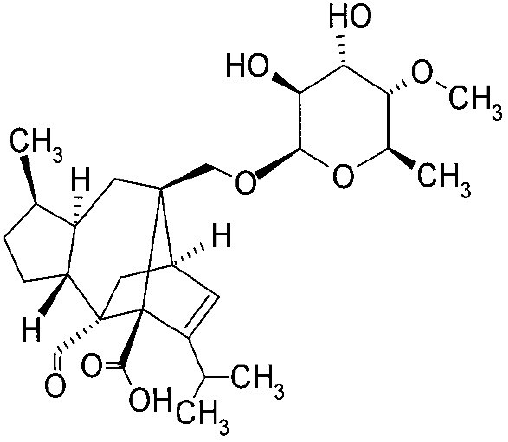  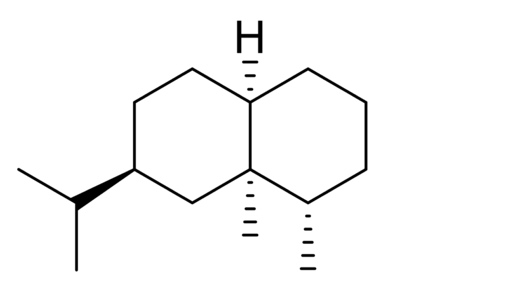 | Antifungal activity (AFA) | (Ibrahim et al., 2020) |
| *Taxus wallachiana* | | *Alternaria sp* | Altersolanol A & Altenusin (quinones and alkaloids)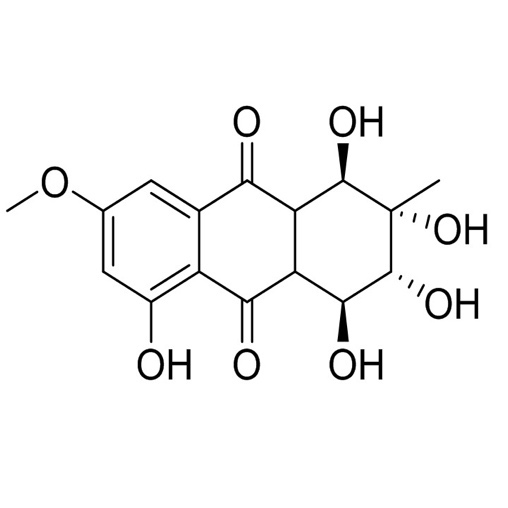  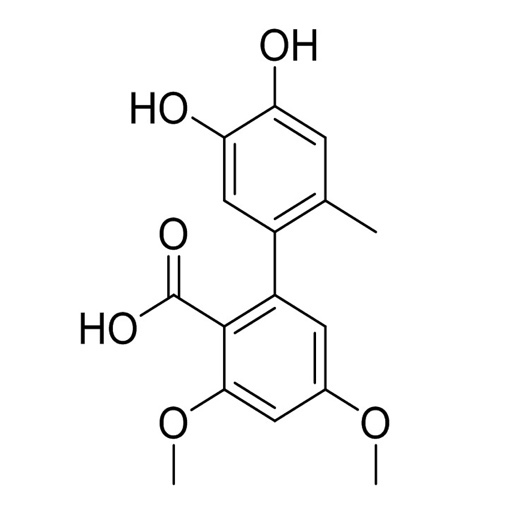 | Antibacterial activity (ABA) | (Anamika et al., 2018; Mousa & Raizada, 2013) |
| *Corylus avellana* | | *Penicillium sps* | VOCs, steroids, paclitaxel  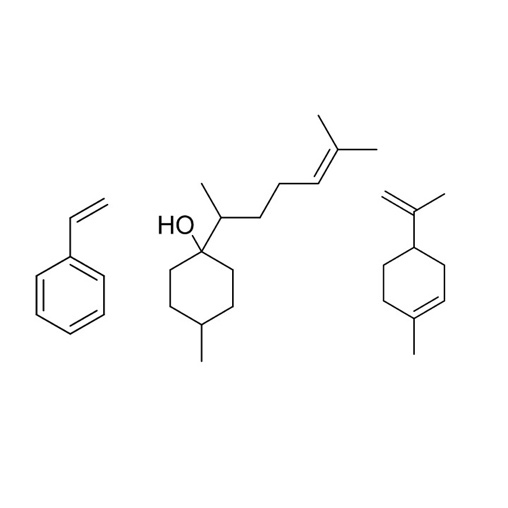 | Antifungal, promotes growth, starch accumulation, anti-cancer | (Anamika et al., 2018; Mousa & Raizada, 2013) |
| *Tripterygium wilfordii* | | *Fusarium sp.* | Terpenoids (diterpenes), VOCs  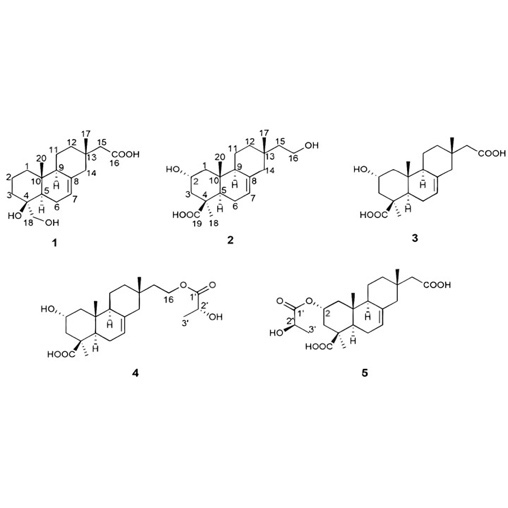 | Promotes growth, anti-microbial | (Anamika et al., 2018) |
| *T. brevifolia* | | *Taxomyces andreanate* | Taxol (Paclitaxel) [diterpenes]  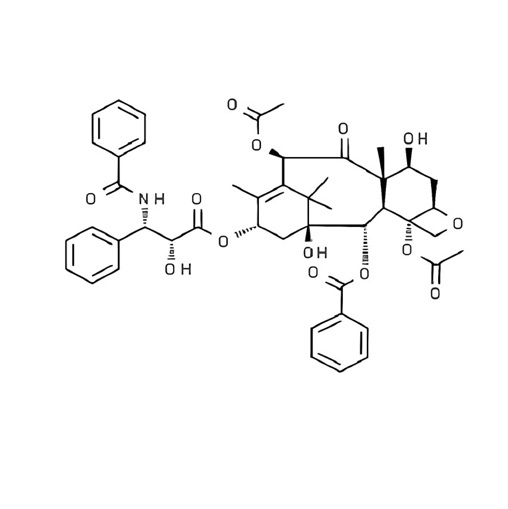 | Anti-fungal activity, Anti-cancer | (Mousa & Raizada, 2013) |
| *Pinus sylvestris*  and *Fagus sylvatica* | | *Aspergillus sp.* | Asporyzin, Cyclopeptides echinocandins (CE) (peptides and diterpenes)  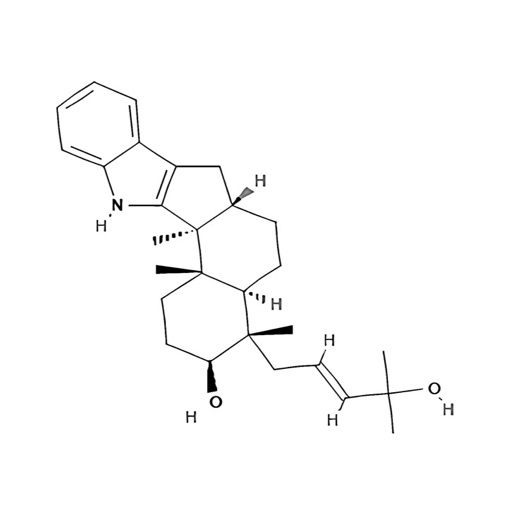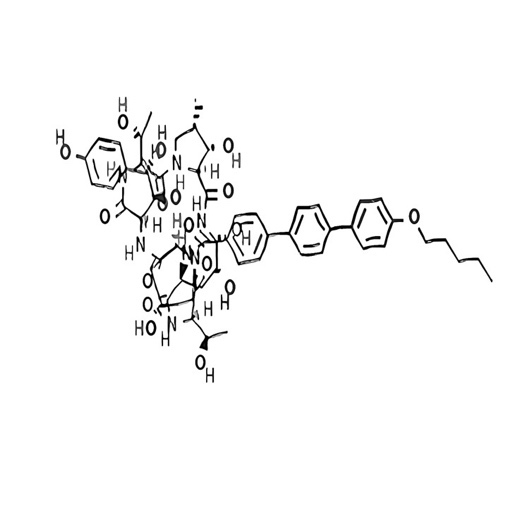 | Anti-fungal, pesticidal | (Anamika et al., 2018; Mousa & Raizada, 2013) |
| *Tripidium arundinaceum* | | *Trichoderma harzianum* | Trichodermin  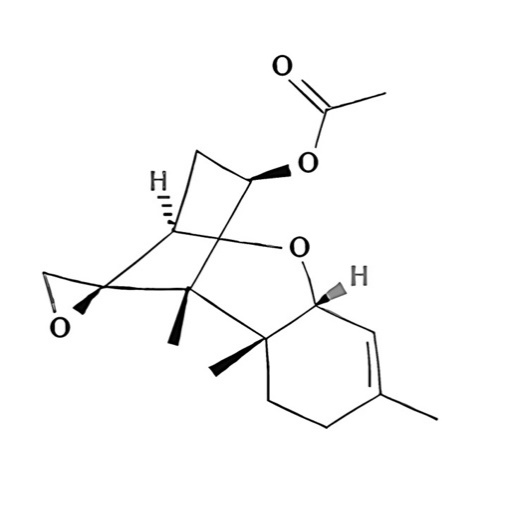 | Anti-fungal, and inhibitory effects, synthesis of PGR | (Mousa & Raizada, 2013) |
| *Urospermum picroides* | | *Ampelomyces sp* | Altersonalol A  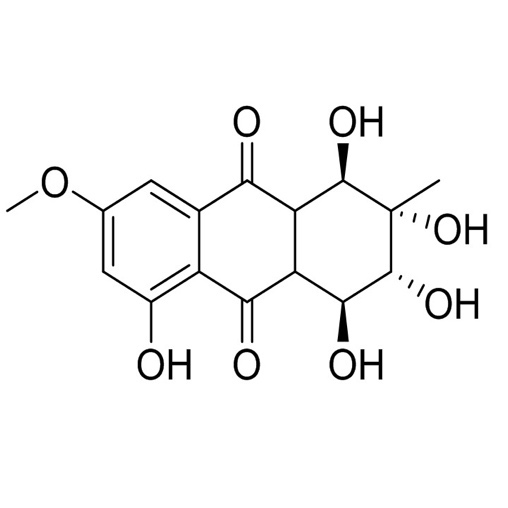 | For the biocontrol of parasitic fungi | (Mousa & Raizada, 2013) |
| *Phleum*pratense | | *E. typhina , Pezicula sp* | CE; (R)-mellein  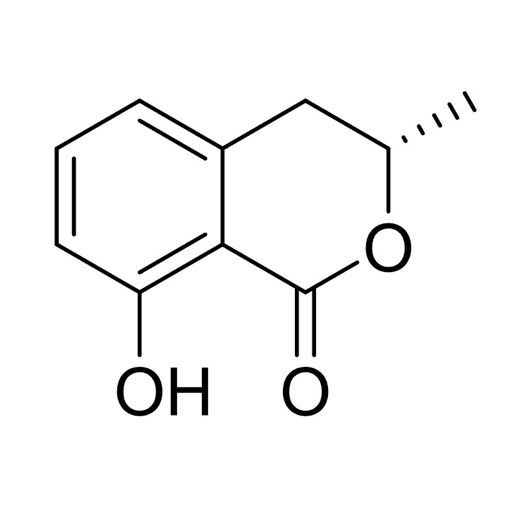 | AMA and larvicidal | (Anamika et al., 2018) |
| *Arisaema erubescens,* Saurauia scaberrinae*Hemsley* | | *Phoma sp.* | Altersolanol A; Phomadecalin C  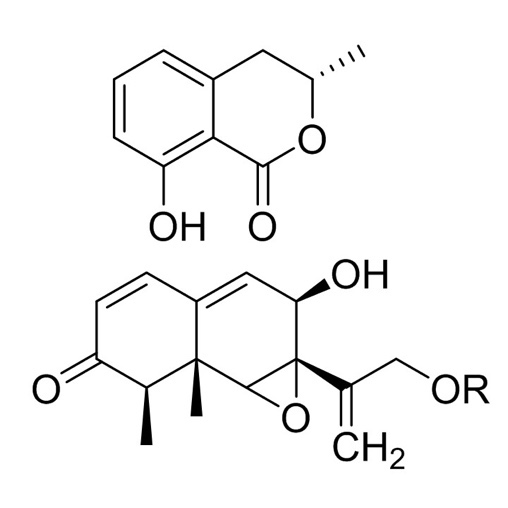 | AMA, antagonistic effect (AGE) | (Anamika et al., 2018) |
| *Bruguiera gymnorrhiza* | | *Cladosporium sp.* | Methyl benzoate; phomaketides, and other VOCs  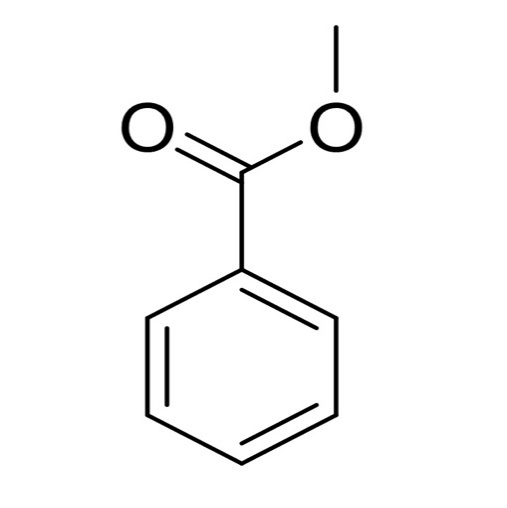 | AMA, AFA | (Anamika et al., 2018) |
| *Lolium perenne* (Perennial ryegrass) | | *Neotyphodium sp.* | Peramine; ergot alkaloids; tricin, clavines (agro, chano and elymo)  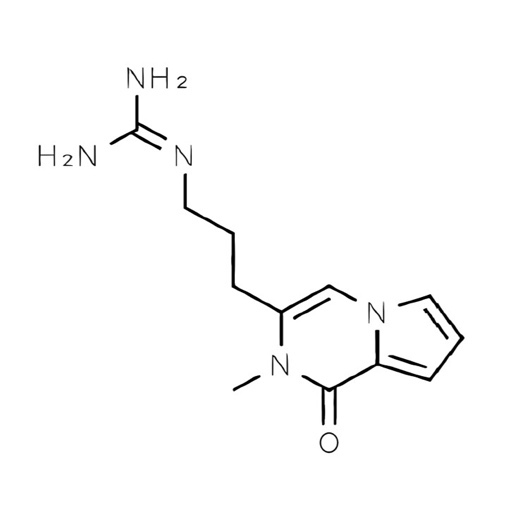  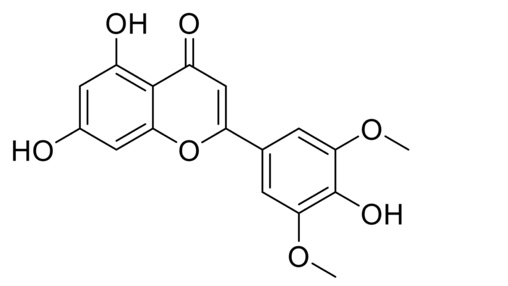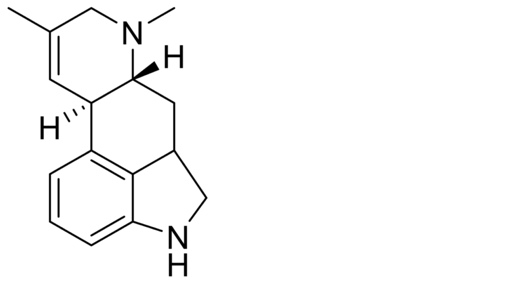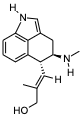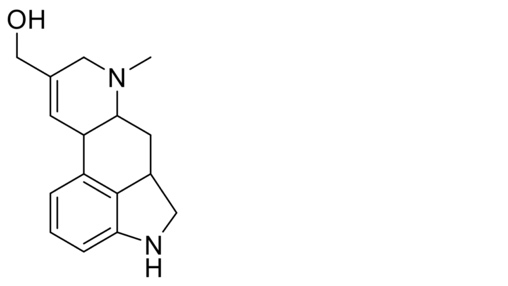 | Pesticidal, AMA, larvicidal | (Anamika et al., 2018; Mousa & Raizada, 2013) |
| *Taxus baccata* | | Acremonium sp. | Leucinostatin A; Peramine (loline alkaloids); pyrrocidines A and B  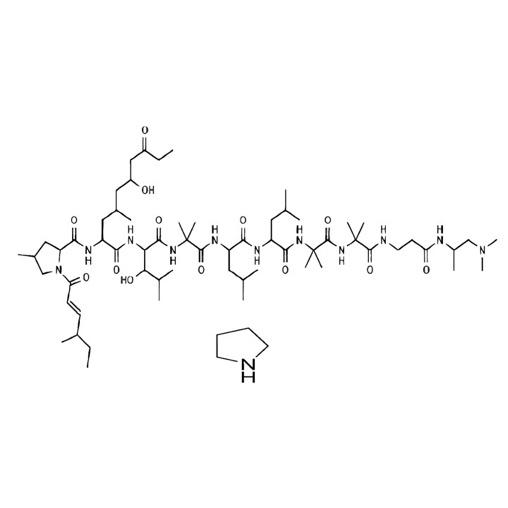 | Phytotoxic, AFA, anti-feedants and antibiotic | (Anamika et al., 2018; Mousa & Raizada, 2013) |
| *Panax notoginseng* | | *T. viridae* | Various types of alcoholics and ketonic compounds, α-ylangene and zingiberene (VOCs)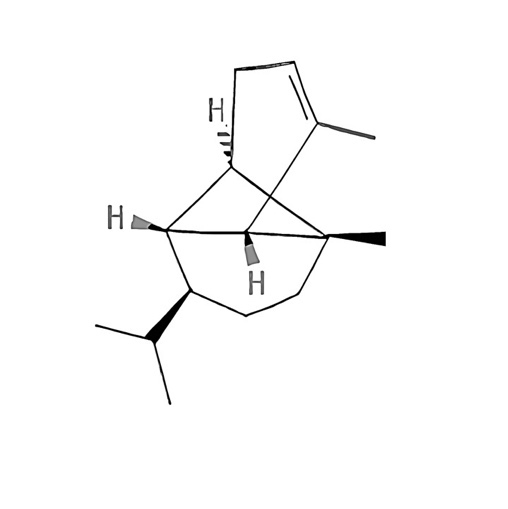  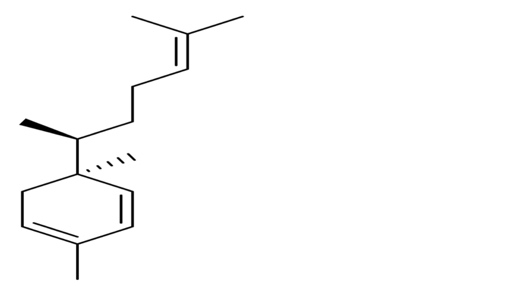 | PGR, development & flowering | (Anamika et al., 2018) |
| *Artemisia annua* | | *Colletotrichum sp.* | 6-isoprenyl indole-3-carboxylic acid and steroids; Colletotric acid  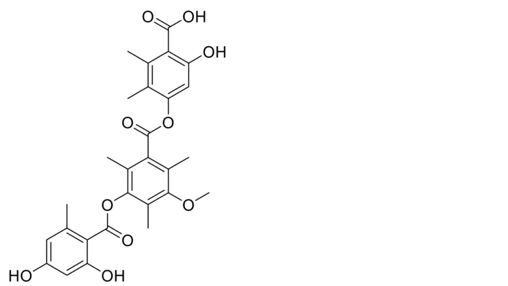  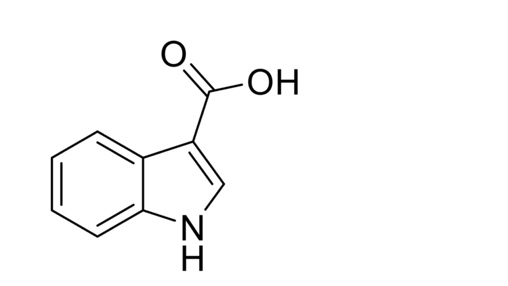 | AFA, ABA, AGE & pesticidal | (Anamika et al., 2018) |
| Lemon grass  (*Cymbopogon citratus*) | | *Phoma, Colletotrichum, Penicillium, Fusarium* | l-asparaginase production | Anticancer activity | (Chow & Ting, 2015) |
| Chicory  (*Cichorium intybus*) | | *Cladosporium sp,*  *Alternaria, Epicoccum* | L-asparaginase  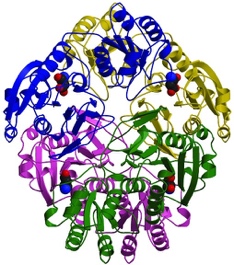 | Anticancer activity | (Hatamzadeh et al., 2020) |

**References**

Abdelshafy Mohamad, O. A., Ma, J. B., Liu, Y. H., Zhang, D., Hua, S., Bhute, S., Hedlund, B. P., Li, W. J., & Li, L. (2020). Beneficial Endophytic Bacterial Populations Associated With Medicinal Plant Thymus vulgaris Alleviate Salt Stress and Confer Resistance to Fusarium oxysporum. *Frontiers in Plant Science*, *11*. https://doi.org/10.3389/fpls.2020.00047

Adrees, H., Adrees, H., Haider, M. S., Anjum, T., & Akram, W. (2019). Inducing systemic resistance in cotton plants against charcoal root rot pathogen using indigenous rhizospheric bacterial strains and chemical elicitors. *Crop Protection*, *v. 115*, 75-83–2019 v.115. https://doi.org/10.1016/j.cropro.2018.09.011

Ai, W., Wei, X., Lin, X., Sheng, L., Wang, Z., Tu, Z., Yang, X., Zhou, X., Li, J., & Liu, Y. (2014). Guignardins A–F, spirodioxynaphthalenes from the endophytic fungus Guignardia sp. KcF8 as a new class of PTP1B and SIRT1 inhibitors. *Tetrahedron*, *70*(35), 5806–5814. https://doi.org/https://doi.org/10.1016/j.tet.2014.06.041

al Ayed, K., Ballantine, R. D., Hoekstra, M., Bann, S. J., Wesseling, C. M. J., Bakker, A. T., Zhong, Z., Li, Y.-X., Brüchle, N. C., van der Stelt, M., Cochrane, S. A., & Martin, N. I. (2022). Synthetic studies with the brevicidine and laterocidine lipopeptide antibiotics including analogues with enhanced properties and in vivo efficacy. *Chemical Science*, *13*(12), 3563–3570. https://doi.org/10.1039/D2SC00143H

Anamika, Joshi, S., Sahgal, M., Sahu, S., & Prakash, A. (2018). Fungal Endophytes and Their Secondary Metabolites: Role in Sustainable Agriculture. In P. Gehlot & J. Singh (Eds.), *Fungi and their Role in Sustainable Development: Current Perspectives* (pp. 121–146). Springer Singapore. https://doi.org/10.1007/978-981-13-0393-7_8

Caruso, G., Abdelhamid, M. T., Kalisz, A., & Sekara, A. (2020). Linking endophytic fungi to medicinal plants therapeutic activity. A case study on asteraceae. In *Agriculture (Switzerland)* (Vol. 10, Issue 7, pp. 1–23). MDPI AG. https://doi.org/10.3390/agriculture10070286

Chow, Y., & Ting, A. S. Y. (2015). Endophytic l-asparaginase-producing fungi from plants associated with anticancer properties. *Journal of Advanced Research*, *6*(6), 869–876. https://doi.org/https://doi.org/10.1016/j.jare.2014.07.005

Conti, R., Chagas, F. O., Caraballo-Rodriguez, A. M., Melo, W. G. da P., do Nascimento, A. M., Cavalcanti, B. C., de Moraes, M. O., Pessoa, C., Costa-Lotufo, L. V., Krogh, R., Andricopulo, A. D., Lopes, N. P., & Pupo, M. T. (2016). Endophytic Actinobacteria from the Brazilian Medicinal Plant Lychnophora ericoides Mart. and the Biological Potential of Their Secondary Metabolites. *Chemistry & Biodiversity*, *13*(6), 727–736. https://doi.org/https://doi.org/10.1002/cbdv.201500225

Costa, L. M., Molina de Olyveira, G., & Salomão, R. (2017). Precipitated Calcium Carbonate Nano-Microparticles: Applications in Drug Delivery. In *Advances in Tissue Engineering and Regenerative Medicine* (Vol. 3). https://doi.org/10.15406/atroa.2017.03.00059

Das, P., Sarkar, B., & Mandal, S. (2023). 21 - Endophytic microbes: A potential source of bioactive metabolites with therapeutic values. In M. Shah & D. Deka (Eds.), *Endophytic Association: What, Why and How* (pp. 435–457). Academic Press. https://doi.org/https://doi.org/10.1016/B978-0-323-91245-7.00004-3

Ding, L., Münch, J., Goerls, H., Maier, A., Fiebig, H.-H., Lin, W.-H., & Hertweck, C. (2010). Xiamycin, a pentacyclic indolosesquiterpene with selective anti-HIV activity from a bacterial mangrove endophyte. *Bioorganic & Medicinal Chemistry Letters*, *20*(22), 6685–6687. https://doi.org/https://doi.org/10.1016/j.bmcl.2010.09.010

Ding, W., Liu, W.-Q., Jia, Y., Li, Y., van der Donk, W. A., & Zhang, Q. (2016). Biosynthetic investigation of phomopsins reveals a widespread pathway for ribosomal natural products in Ascomycetes. *Proceedings of the National Academy of Sciences*, *113*(13), 3521–3526. https://doi.org/10.1073/pnas.1522907113

Ezra, D., Castillo, U. F., Strobel, G. A., Hess, W. M., Porter, H., Jensen, J. B., Condron, M. A. M., Teplow, D. B., Sears, J., Maranta, M., Hunter, M., Weber, B., & Yaver, D. (2004). Coronamycins, peptide antibiotics produced by a verticillate Streptomyces sp. (MSU-2110) endophytic on Monstera sp. *Microbiology*, *150*(4), 785–793. https://doi.org/10.1099/mic.0.26645-0

Fu, Y., Yin, Z.-H., & Yin, C.-Y. (2017). Biotransformation of ginsenoside Rb1 to ginsenoside Rg3 by endophytic bacterium Burkholderia sp. GE 17-7 isolated from Panax ginseng. *Journal of Applied Microbiology*, *122*(6), 1579–1585. https://doi.org/https://doi.org/10.1111/jam.13435

Ghiasvand, M., Makhdoumi, A., Matin, M. M., & Vaezi, J. (2020). Exploring the bioactive compounds from endophytic bacteria of a medicinal plant: Ephedra foliata (Ephedrales: Ephedraceae). *Advances in Traditional Medicine*, *20*(1), 61–70. https://doi.org/10.1007/s13596-019-00410-z

Gos, F. M. W. R., Savi, D. C., Shaaban, K. A., Thorson, J. S., Aluizio, R., Possiede, Y. M., Rohr, J., & Glienke, C. (2017). Antibacterial activity of endophytic actinomycetes isolated from the medicinal plant Vochysia divergens (Pantanal, Brazil). *Frontiers in Microbiology*, *8*(SEP). https://doi.org/10.3389/fmicb.2017.01642

Hatamzadeh, S., Rahnama, K., Nasrollahnejad, S., Fotouhifar, K. B., Hemmati, K., White, J. F., & Taliei, F. (2020). Isolation and identification of L-asparaginase-producing endophytic fungi from the Asteraceae family plant species of Iran. *PeerJ*, *2020*(1). https://doi.org/10.7717/peerj.8309

Huang, Y. (2019). Illumina-based Analysis of Endophytic Bacterial Diversity of four Allium species. *Scientific Reports*, *9*(1), 15271. https://doi.org/10.1038/s41598-019-51707-7

Ibrahim, A., Tanney, J. B., Fei, F., Seifert, K. A., Cutler, G. C., Capretta, A., Miller, J. D., & Sumarah, M. W. (2020). Metabolomic-guided discovery of cyclic nonribosomal peptides from Xylaria ellisii sp. nov., a leaf and stem endophyte of Vaccinium angustifolium. *Scientific Reports*, *10*(1). https://doi.org/10.1038/s41598-020-61088-x

Jain, S., Vaishnav, A., Kasotia, A., Kumari, S., Gaur, R. K., & Choudhary, D. K. (2014). Rhizobacterium-mediated growth promotion and expression of stress enzymes in Glycine max L. Merrill against Fusarium wilt upon challenge inoculation. *World Journal of Microbiology and Biotechnology*, *30*(2), 399–406. https://doi.org/10.1007/s11274-013-1455-5

Jia, M., Chen, L., Xin, H. L., Zheng, C. J., Rahman, K., Han, T., & Qin, L. P. (2016). A friendly relationship between endophytic fungi and medicinal plants: A systematic review. In *Frontiers in Microbiology* (Vol. 7, Issue JUN). Frontiers Research Foundation. https://doi.org/10.3389/fmicb.2016.00906

Kusari, S., Lamshöft, M., & Spiteller, M. (2009). Aspergillus fumigatus Fresenius, an endophytic fungus from Juniperus communis L. Horstmann as a novel source of the anticancer pro-drug deoxypodophyllotoxin. *Journal of Applied Microbiology*, *107*(3), 1019–1030. https://doi.org/https://doi.org/10.1111/j.1365-2672.2009.04285.x

Liu, Y., Liu, W., & Liang, Z. (2015). Endophytic bacteria from Pinellia ternata, a new source of purine alkaloids and bacterial manure. *Pharmaceutical Biology*, *53*(10), 1545–1548. https://doi.org/10.3109/13880209.2015.1016580

Mousa, W. K., & Raizada, M. N. (2013). The diversity of anti-microbial secondary metabolites produced by fungal endophytes: an interdisciplinary perspective. In *Frontiers in Microbiology* (Vol. 4). Frontiers Media S.A. https://doi.org/10.3389/fmicb.2013.00065

Parthasarathy, R., & Sathiyabama, M. (2015). Lovastatin-producing endophytic fungus isolated from a medicinal plant Solanum xanthocarpum. *Natural Product Research*, *29*(24), 2282–2286. https://doi.org/10.1080/14786419.2015.1016938

Peng, R., Xu, Y., Zhu, T., Li, N., Qi, J., Chai, Y., Wu, M., Zhang, X., Shi, Y., Wang, P., Wang, J., Gao, N., & Gao, G. F. (2017). Alternate binding modes of anti-CRISPR viral suppressors AcrF1/2 to Csy surveillance complex revealed by cryo-EM structures. *Cell Research*, *27*(7), 853–864. https://doi.org/10.1038/cr.2017.79

Qin, J.-C., Zhang, Y.-M., Gao, J.-M., Bai, M.-S., Yang, S.-X., Laatsch, H., & Zhang, A.-L. (2009). Bioactive metabolites produced by Chaetomium globosum, an endophytic fungus isolated from Ginkgo biloba. *Bioorganic & Medicinal Chemistry Letters*, *19*(6), 1572–1574. https://doi.org/https://doi.org/10.1016/j.bmcl.2009.02.025

Sabu, R., Soumya, K. R., & Radhakrishnan, E. K. (2017). Endophytic Nocardiopsis sp. from Zingiber officinale with both antiphytopathogenic mechanisms and antibiofilm activity against clinical isolates. *3 Biotech*, *7*(2), 115. https://doi.org/10.1007/s13205-017-0735-4

Singh, R., & Dubey, A. K. (2018). Diversity and applications of endophytic actinobacteria of plants in special and other ecological niches. In *Frontiers in Microbiology* (Vol. 9, Issue AUG). Frontiers Media S.A. https://doi.org/10.3389/fmicb.2018.01767

Strobel, G., Daisy, B., Castillo, U., & Harper, J. (2004). Natural Products from Endophytic Microorganisms. *Journal of Natural Products*, *67*(2), 257–268. https://doi.org/10.1021/np030397v

Swamy, M. K., Das, T., Nandy, S., Mukherjee, A., Pandey, D. K., & Dey, A. (2022). 8 - Endophytes for the production of anticancer drug, paclitaxel. In M. K. Swamy, T. Pullaiah, & Z.-S. Chen (Eds.), *Paclitaxel* (pp. 203–228). Academic Press. https://doi.org/https://doi.org/10.1016/B978-0-323-90951-8.00012-6

Tiwari, P., Kang, S., & Bae, H. (2023). Plant-endophyte associations: Rich yet under-explored sources of novel bioactive molecules and applications. *Microbiological Research*, *266*, 127241. https://doi.org/https://doi.org/10.1016/j.micres.2022.127241

Toghueo, R. M. K. (2020). Endophytes from Gingko biloba: the current status. *Phytochemistry Reviews*, *19*(4), 743–759. https://doi.org/10.1007/s11101-020-09679-4

Uzma, F., & Chowdappa, S. (2021). Chapter 12 - Endophytic fungal metabolites of medicinal plants and their bioactive properties. In J. Singh & P. Gehlot (Eds.), *New and Future Developments in Microbial Biotechnology and Bioengineering* (pp. 165–176). Elsevier. https://doi.org/https://doi.org/10.1016/B978-0-12-821005-5.00012-0

Wang, Y., Zeng, Q. G., Zhang, Z. bin, Yan, R. M., Wang, L. Y., & Zhu, D. (2011). Isolation and characterization of endophytic huperzine A-producing fungi from Huperzia serrata. *Journal of Industrial Microbiology and Biotechnology*, *38*(9), 1267–1278. https://doi.org/10.1007/s10295-010-0905-4

Wen, J., Okyere, S. K., Wang, S., Wang, J., Xie, L., Ran, Y., & Hu, Y. (2022). Endophytic Fungi: An Effective Alternative Source of Plant‐Derived Bioactive Compounds for Pharmacological Studies. In *Journal of Fungi* (Vol. 8, Issue 2). MDPI. https://doi.org/10.3390/jof8020205

Yan, H., Jin, H., Fu, Y., Yin, Z., & Yin, C. (2019a). Production of Rare Ginsenosides Rg3 and Rh2 by Endophytic Bacteria from Panax ginseng. *Journal of Agricultural and Food Chemistry*, *67*(31), 8493–8499. https://doi.org/10.1021/acs.jafc.9b03159

Yan, H., Jin, H., Fu, Y., Yin, Z., & Yin, C. (2019b). Production of Rare Ginsenosides Rg3 and Rh2 by Endophytic Bacteria from Panax ginseng. *Journal of Agricultural and Food Chemistry*, *67*(31), 8493–8499. https://doi.org/10.1021/acs.jafc.9b03159

Yan, Y., Zhao, J., Cao, C., Jia, Z., Zhou, N., Han, S., Wang, Y., Xu, Y., Zhao, J., Yan, Y., & Cui, H. (2014). Tetramethylpyrazine promotes SH-SY5Y cell differentiation into neurons through epigenetic regulation of Topoisomerase IIβ. *Neuroscience*, *278*, 179–193. https://doi.org/https://doi.org/10.1016/j.neuroscience.2014.08.010

Yin, D. D., Wang, Y. L., Yang, M., Yin, D. K., Wang, G. K., & Xu, F. (2019). Analysis of Chuanxiong Rhizoma substrate on production of ligustrazine in endophytic Bacillus subtilis by ultra high performance liquid chromatography with quadrupole time-of-flight mass spectrometry. *Journal of Separation Science*, *42*(19), 3067–3076. https://doi.org/https://doi.org/10.1002/jssc.201900030

Zhang, G., Sun, S., Zhu, T., Lin, Z., Gu, J., Li, D., & Gu, Q. (2011). Antiviral isoindolone derivatives from an endophytic fungus Emericella sp. associated with Aegiceras corniculatum. *Phytochemistry*, *72*(11), 1436–1442. https://doi.org/https://doi.org/10.1016/j.phytochem.2011.04.014

Zhang, J. Y., Tao, L. Y., Liang, Y. J., Chen, L. M., Mi, Y. J., Zheng, L. S., Wang, F., She, Z. G., Lin, Y. C., To, K. K. W., & Fu, L. W. (2010). Anthracenedione derivatives as anticancer agents isolated from secondary metabolites of the mangrove endophytic fungi. *Marine Drugs*, *8*(4), 1469–1481. https://doi.org/10.3390/md8041469
